# Low-input redoxomics facilitates global identification of metabolic regulators of oxidative stress in the gut

**DOI:** 10.1038/s41392-024-02094-7

**Published:** 2025-01-08

**Authors:** Xina Xiao, Meng Hu, Li Gao, Huan Yuan, Baochen Chong, Yu Liu, Rou Zhang, Yanqiu Gong, Dan Du, Yong Zhang, Hao Yang, Xiaohui Liu, Yan Zhang, Huiyuan Zhang, Heng Xu, Yi Zhao, Wenbo Meng, Dan Xie, Peng Lei, Shiqian Qi, Yong Peng, Tao Tan, Yang Yu, Hongbo Hu, Biao Dong, Lunzhi Dai

**Affiliations:** 1 National Clinical Research Center for Geriatrics, State Key Laboratory of Biotherapy, West China Hospital, Sichuan University, Chengdu, China; 2 Advanced Mass Spectrometry Center, Research Core Facility, Frontiers Science Center for Disease-Related Molecular Network, NHC Key Lab of Transplant Engineering and Immunology, West China Hospital, Sichuan University, Chengdu, China; 3 School of Life Sciences, Tsinghua University, Beijing, China; 4 Department of Rheumatology and Immunology, West China Hospital, Sichuan University, Chengdu, China; 5 The First School of Clinical Medicine, Lanzhou University, Lanzhou, China; 6 State Key Laboratory of Primate Biomedical Research, Institute of Primate Translational Medicine, Kunming University of Science and Technology, Kunming, China; 7 Beijing Key Laboratory of Reproductive Endocrinology and Assisted Reproductive Technology and Key Laboratory of Assisted Reproduction, Ministry of Education, Center for Reproductive Medicine, Department of Obstetrics and Gynecology, Peking University Third Hospital, Beijing, China; 8 Frontiers Medical Center, Tianfu Jincheng Laboratory, Chengdu, China

**Keywords:** Ageing, Biological techniques

## Abstract

Oxidative stress plays a crucial role in organ aging and related diseases, yet the endogenous regulators involved remain largely unknown. This work highlights the importance of metabolic homeostasis in protecting against oxidative stress in the large intestine. By developing a low-input and user-friendly pipeline for the simultaneous profiling of five distinct cysteine (Cys) states, including free SH, total Cys oxidation (Sto), sulfenic acid (SOH), *S*-nitrosylation (SNO), and *S*-glutathionylation (SSG), we shed light on Cys redox modification stoichiometries and signaling with regional resolution in the aging gut of monkeys. Notably, the proteins modified by SOH and SSG were associated primarily with cell adhesion. In contrast, SNO-modified proteins were involved in immunity. Interestingly, we observed that the Sto levels ranged from 0.97% to 99.88%, exhibiting two distinct peaks and increasing with age. Crosstalk analysis revealed numerous age-related metabolites potentially involved in modulating oxidative stress and Cys modifications. Notably, we elucidated the role of fumarate in alleviating intestinal oxidative stress in a dextran sulfate sodium (DSS)-induced colitis mouse model. Our findings showed that fumarate treatment promotes the recovery of several cell types, signaling pathways, and genes involved in oxidative stress regulation. Calorie restriction (CR) is a known strategy for alleviating oxidative stress. Two-month CR intervention led to the recovery of many antioxidative metabolites and reshaped the Cys redoxome. This work decodes the complexities of redoxomics during the gut aging of non-human primates and identifies key metabolic regulators of oxidative stress and redox signaling.

## Introduction

The large intestine, including the colon and rectum, is a complex organ populated with a diverse community of microorganisms.^1^ As individuals age, the likelihood of experiencing oxidative stress in the gut tends to increase.^2^ Free radicals contributing to intestinal oxidative stress can originate from various sources, including normal cellular metabolism, inflammatory processes, dietary factors, environmental exposures, and gut microbiota.^3,4^ Aged-related oxidative stress coupled with a functional decline in antioxidant defense systems^5,6^ can have detrimental effects on barrier integrity, gut microbiota, and immune function.^7–10^

Protein cysteine (Cys) modifications, such as sulfenic acid (SOH), sulfinic acid (SO_2_H), sulfonic acid (SO_3_H), *S*-glutathionylation (SSG), *S*-nitrosylation (SNO), and disulfide bonds (S‒S), are closely linked to oxidative stress and form a dynamic regulatory network within cells.^11^ These post-translational modifications (PTMs), along with the unoxidized state (SH) of Cys residues, collectively contribute to the overall redox balance in cells and organs.^12–18^ Imbalances in these modifications not only serve as indicators of oxidative stress but also may promote oxidative damage within the gut, potentially contributing to gut aging and disease conditions.^19^ However, to date, systematic exploration of their landscapes, stoichiometric, and functions during gut aging, especially in primates, is still lacking.^5^ Furthermore, the endogenous regulators of oxidative stress and Cys modifications remain largely unknown.

High-throughput profiling of Cys redox modifications in proteins is crucial for understanding how oxidative stress and redox signaling impact cellular processes.^20–22^ A key step in redoxomics analysis is the selective reduction of Cys modifications. Several methods have been developed for this purpose.^23^ For example, the total Cys oxidation (Sto) state, including SOH, SO_2_H, SO_3_H, SSG, SNO, and S‒S, can be reduced via tris(2-carboxyethyl)phosphine (TCEP).^24^ Additionally, the selective reduction of SNO to free Cys is achieved via ascorbate, whereas SOH is reduced by arsenite.^25^ Furthermore, the conversion of SSG to free Cys can be catalyzed by enzymes such as glutaredoxin (Grx).^26^ Following selective reduction, an isobaric labeling strategy enables the quantification of alterations in total oxidation and specific redox modifications.^24–27^ Remarkably, several highly chemoselective probes have been developed to label Cys redox forms (SH, SOH, and SO_2_H).^28,29^ Despite tremendous progress, several aspects demand further enhancement, including chemical labeling selectivity and efficiency, as well as enrichment and elution efficiency. Moreover, the need for a large sample input and a substantial amount of isotopic labeling reagents in the current strategies limit widespread application.

**Fig. 1:**
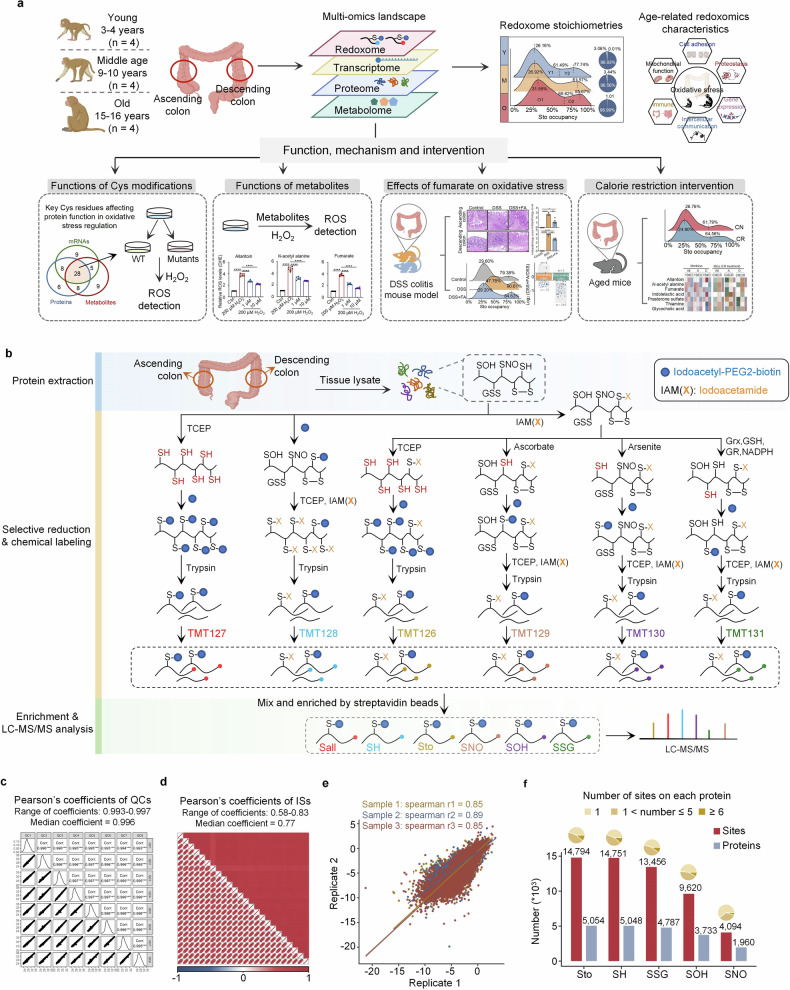
Characterization of Cys redoxomics associated with gut aging. **a** Schematic of multi-omics analysis of intestinal aging in non-human primates. **b** Flow chart of high-throughput Cys redoxomics analysis, covering free Cys (SH), sulfenic acid (SOH), *S*-glutathionylation (SSG), *S*-nitrosylation (SNO), and total oxidation (Sto). The blue circle signifies iodoacetyl-PEG2-biotin, and X represents iodoacetamide (IAM). **c** Pearson’s correlation analysis of eight QC samples. **d** Pearson’s correlation analysis of common reference samples. **e** Spearman’s correlation analysis of technical replicates for three representative samples. **f** Statistics of five Cys redox modification states. The bar graph shows the number of sites (red) and proteins (blue). The pie chart shows the number distribution of modified Cys residues on each protein. The elements in (**a**, **b**) were created via BioRender (https://biorender.com)

This work aims to elucidate Cys redox signaling in the aging gut of non-human primates and to identify the endogenous regulators of oxidative stress. To achieve this goal, we developed an efficient pipeline for simultaneously profiling five distinct redox states of Cys residues using only 60 µg of total peptides for isobaric labeling. Using this strategy, we revealed proteome-wide Cys redox signaling associated with gut aging in non-human primates (*Macaca fascicularis*). Moreover, we identified many age-related metabolites, particularly fumarate, as regulators of oxidative stress. Additionally, we demonstrated that calorie restriction (CR) affects oxidative stress and Cys redox signaling in the guts of aged mice, accompanied by the recovery of many antioxidative metabolites (Fig. 1a).

## Results

### Characterization of Cys redox modifications with low protein inputs

To study the role of Cys redox signaling in normal gut aging in higher animals, we collected 24 fresh tissue samples from the ascending and descending colons of 12 female *Macaca fascicularis* individuals. Among these monkeys, 4 were young (3–4 years old), 4 were middle-aged (9–10 years old), and 4 were aged (15–16 years old) (Fig. 1a and Supplementary Table 1a). By applying a strategy that relies on biotin probe labeling followed by tandem mass tag (TMT) labeling, we simultaneously quantified five distinct Cys redox states (free SH, Sto, SOH, SNO, and SSG) (Fig. 1b). The reduction of each Cys modification was performed in accordance with well-established methods as previously described.^24–26^ Specifically, for free protein thiol groups, iodoacetyl-PEG2-biotin was used directly for alkylation and biotin labeling. For other oxidation states, iodoacetamide (IAM) was first employed to block the free thiol groups, after which the samples were treated with different reducing agents. Sodium ascorbate and sodium arsenite were used as reducing agents for SNO and SOH modifications, respectively. For SSG, Grx, glutathione reductase (GR), oxidized glutathione (GSSG), and NADPH were utilized. After reduction to thiol groups, these cysteine residues were rapidly blocked with iodoacetyl-PEG2-biotin. Combining this method with TMT labeling allows for clear differentiation of samples treated with various methods. Besides, a common reference sample generated by TCEP was included in each TMT batch. Notably, as little as 10 µg of peptide input into each plex of TMT reagents is sufficient for profiling purposes. The stability of the machines and the high quality of the redoxome data were confirmed by the reproducibility of the continuous quality control (QC) samples (Fig. 1c and Supplementary Table 1b), the inter-plex common reference (Fig. 1d and Supplementary Table 1c) and the replicate samples (Fig. 1e and Supplementary Table 1d). As a result, we effectively quantified the modification states of 14,811 Cys residues on 5057 proteins with localization probabilities greater than 0.75 by mass spectrometry (MS) (Supplementary Table 1e). Specifically, our redoxomics quantified the SH state of 14,751 Cys residues, the Sto state of 14,794 Cys residues, the SOH state of 9620 Cys residues, the SNO state of 4094 Cys residues, and the SSG state of 13,456 Cys residues in both the ascending and descending colons of non-human primates (Fig. 1f and Supplementary Table 1f).

### Cys redox modification stoichiometry and signatures during gut aging

**Fig. 2:**
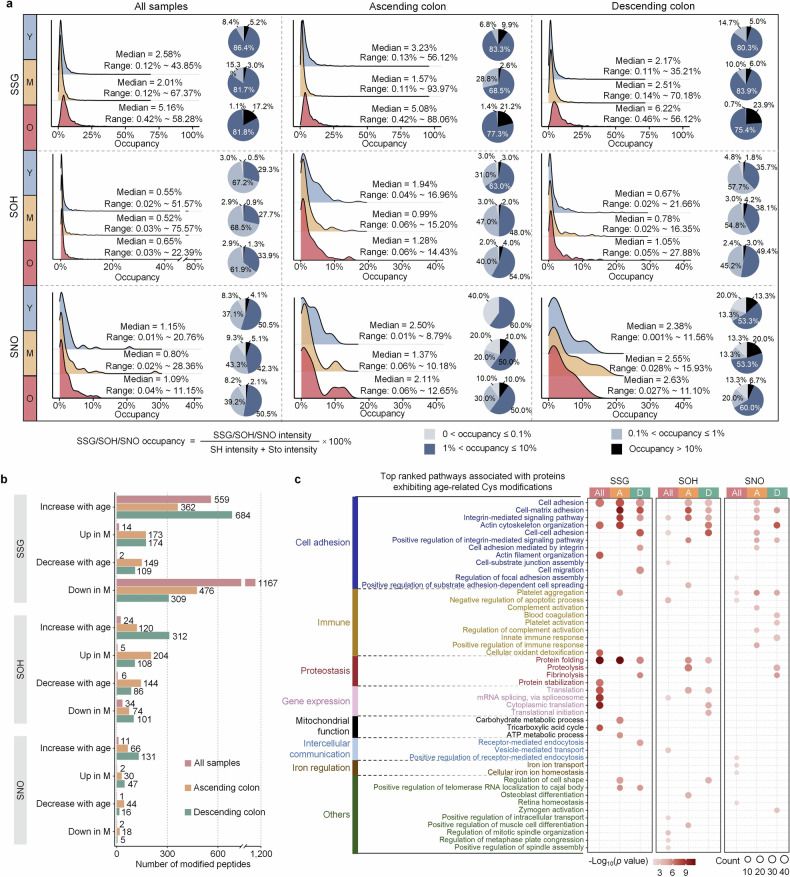
Age- and location-resolved stoichiometries and signatures of SSG, SOH, and SNO modifications. **a** Density plots illustrating the distributions of SSG, SOH, and SNO occupancies with or without consideration of location effects. The occupancy is the median value of the samples. The pie charts display the SSG (or SOH, SNO) occupancy proportion within each age group. Y young, M middle-aged, O old. **b** Statistics of peptides with SSG, SOH, and SNO modifications showing significant differences with age, with or without considering location effects. If modifications were detected in at least three samples from each of the young, middle-aged, and old groups, one-way ANOVA was used (*p* < 0.05). Additionally, if the modification is not detected in one or two of the three groups but is detected in at least half of the samples in the remaining group(s), these modification sites are also defined as differential. Up in M means highest abundance in the middle-age, and Down in M means lowest abundance in the middle-age. Young, M middle-aged, O old. **c** Bubble chart showing the enriched Gene Ontology Biological Process (GOBP) using proteins with significantly changed SSG, SOH, and SNO modifications. These analyses include data without considering location effects (All), as well as data specifically from the ascending colon (A) and descending colon (D). Each group highlights the ten most significantly enriched pathways

Initially, we calculated the stoichiometries and explored the potential effects of SOH, SSG, and SNO modifications associated with gut aging. Statistical analysis revealed a significant increase in the average levels of SOH, SSG, and SNO in aged individuals, both in the ascending and descending colons (Fig. 2a and Supplementary Table 2a–d). The stoichiometries of these three types of redox modifications were relatively low, with median values ranging from 1.57% to 6.22% for SSG, 0.52% to 1.94% for SOH, and 0.80% to 2.63% for SNO. Notably, the level of SSG dramatically increased in aged monkeys, with a median value of 5.08% in the ascending colon and 6.22% in the descending colon, suggesting that SSG modification may be more closely associated with gut aging than SOH and SNO (Fig. 2a). Pathway enrichment analysis of proteins with age-related SOH, SSG, and SNO modification sites revealed the involvement of SOH- and SSG-modified proteins in cell adhesion and protein folding, whereas SNO-modified proteins potentially play crucial roles in the regulation of immunity (Fig. 2b, c and Supplementary Table 2e, f). Furthermore, our findings disclosed location-specific Cys redox regulation associated with gut aging. Age-related SSG-modified proteins were enriched in the cell‒cell adhesion pathway in the descending colon but not in the ascending colon. Immune-associated SNO-modified proteins were involved in distinct pathways between the ascending and descending colon, with pathways such as positive regulation of immune response, complement activation, and regulation of complement activation enriched in the ascending colon. In contrast, the descending colon exhibited specific enrichment of pathways related to blood coagulation, innate immune response, and platelet activation (Fig. 2c). Overall, we present the signatures and stoichiometries of SOH, SSG, and SNO modifications associated with non-human primate gut aging.

### Age-related shifts in the oxidation occupancy of Cys residues

**Fig. 3:**
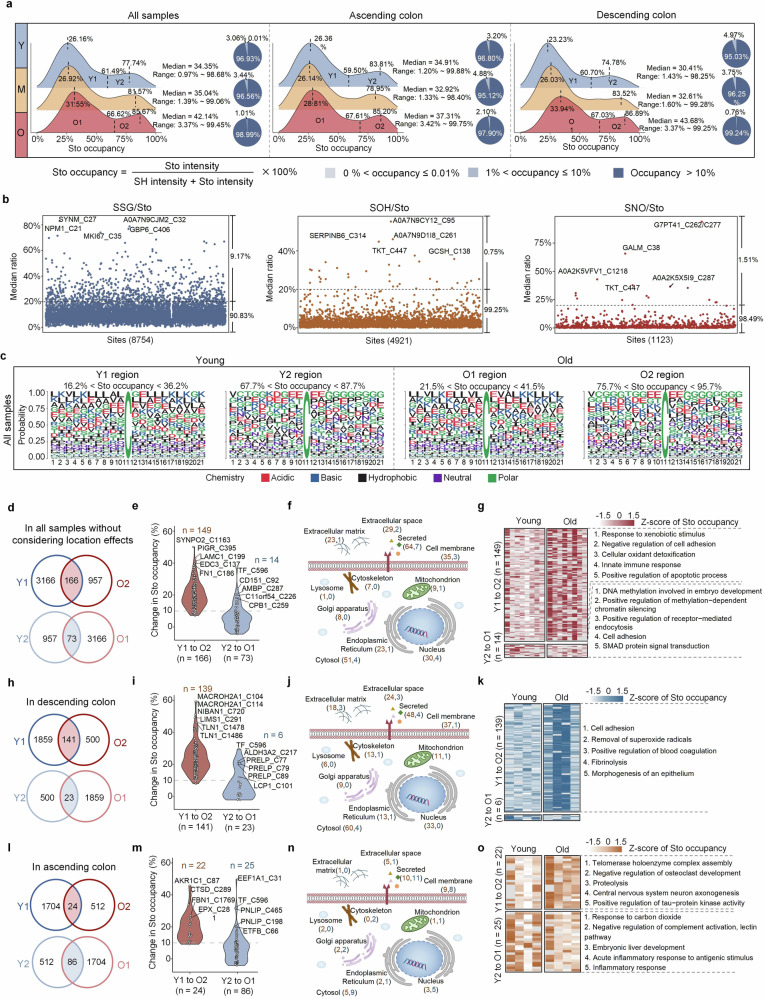
Bimodal characteristics of Cys oxidation occupancy. **a** Density plot illustrating the Sto occupancy distribution with or without considering location effects. The occupancy is the median value of the samples. The pie charts display the Sto occupancy proportion within each age group. The Sto occupancy displays a bimodal distribution, with peaks in the young labeled Y1 and Y2 and peaks in the old labeled O1 and O2. Y young, M middle-aged, O old. **b** Contribution of SSG, SOH, and SNO modifications to the Sto occupancy of each Cys residue. **c** Amino acid motif analysis of the surrounding Cys modification sites in each region. The ‘ggseqlogo’ package in R was used for this analysis. **d**–**o** Age- and location-resolved Cys oxidation characteristics without considering location effects (**d**–**g**) or in the descending colon (**h**–**k**) or ascending colon (**l**–**o**). The number of Cys residues with Sto occupancy transiting from Y1 to O2 (red shading) or from Y2 to O1 (blue shading) (**d**, **h**, and **l**), as well as the number of Cys residues with Sto occupancy changes greater than 10% is shown (**e**, **i**, and **m**). Subcellular localization of proteins with Sto occupancy changes on Cys residues greater than 10% in all samples without considering location effects (**f**), in the descending colon (**j**) and in the ascending colon (**n**) are displayed. The red numbers indicate the number of Cys residues with Sto occupancy transitioning from Y1 to O2, and the blue numbers indicate the number of Cys residues with Sto occupancy transitioning from Y2 to O1 group (**f**, **j**, and **n**). The heatmaps show the enriched Gene Ontology Biological Process (GOBP) using proteins with Sto occupancy changes greater than 10% in all samples without considering location effects (**g**), in the descending colon (**k**) or in the ascending colon (**o**)

Disruption of the Cys redox balance may have a substantial effect on diverse cellular processes and activities, including protein homeostasis and enzyme activities.^30^ Interestingly, our study revealed variations in the Sto occupancy of Cys residues from 3.37% to 99.45%, with two distinct peaks at 31.55% and 85.67% in aged individuals (Fig. 3a and Supplementary Table 3a, b). The median values of Sto occupancy showed an age-related elevation, particularly in the descending colon. Specifically, the first occupancy peak shifted from 23.23% to 33.94%, and the second peak shifted from 74.78% to 86.89% with age (Fig. 3a), which was consistent with the age-related increase in oxidative stress in the aging gut. Interestingly, age-related increases in Cys oxidation were not detected in *Drosophila melanogaster*,^31^ suggesting a species-specific difference. Using the valley position between the two peaks as a boundary, the Cys residues were allocated into two regions on the basis of their oxidation levels. The two regions in young monkeys were labeled Y1 and Y2, whereas the two regions in aged monkeys were termed O1 and O2 (Fig. 3a). We found that, among the SOH, SSG, and SNO modifications, SSG contributed the most to Sto occupancy (Fig. 3b and Supplementary Table 3c). Notably, analysis of amino acid motifs surrounding Cys residues revealed a greater incidence of hydrophobic amino acids (AAs), such as leucine (L), and basic AAs, such as lysine (K), at low Sto occupancy regions (Y1 and O1). Conversely, glycine (G) and glutamic acid (E) were more commonly observed at the second peaks (Y2 and O2) (Fig. 3c and Supplementary Table 3d).

To gain deeper insights into the age-related shifts in Cys oxidation between the two regions, we performed overlap analysis and identified 166 Cys residues that exhibited shifts in Sto occupancy from the Y1 region to the O2 region, indicating elevated oxidation levels with age (Fig. 3d and Supplementary Table 3e). Among these 166 Cys residues, 149 presented more than a 10% increase (Fig. 3e), with the majority being secreted and cytosolic proteins (Fig. 3f and Supplementary Table 3e), and the related proteins might play roles in pathways such as the response to xenobiotic stimulus, negative regulation of cell adhesion and cellular oxidant detoxification (Fig. 3g and Supplementary Table 3e). In contrast, 73 Cys sites exhibited decreased Sto occupancy in aged individuals, transitioning from the Y2 region to the O1 region (Fig. 3d and Supplementary Table 3e), with only 14 showing a greater than 10% reduction (Fig. 3e and Supplementary Table 3e). Similar overlap analyses were carried out for the descending (Fig. 3h–k and Supplementary Table 3f) and ascending (Fig. 3l–o and Supplementary Table 3g) colons. Unexpectedly, our findings revealed that significant age-related increases (Y1 to O2) in Sto occupancy of over 10% occurred mostly in the descending colon (Fig. 3i), whereas notable decreases (Y2 to O1) of over 10% were more evident in the ascending colon (Fig. 3m). These observations suggest that age-related oxidative stress may have a more pronounced effect on the descending colon.

**Fig. 4:**
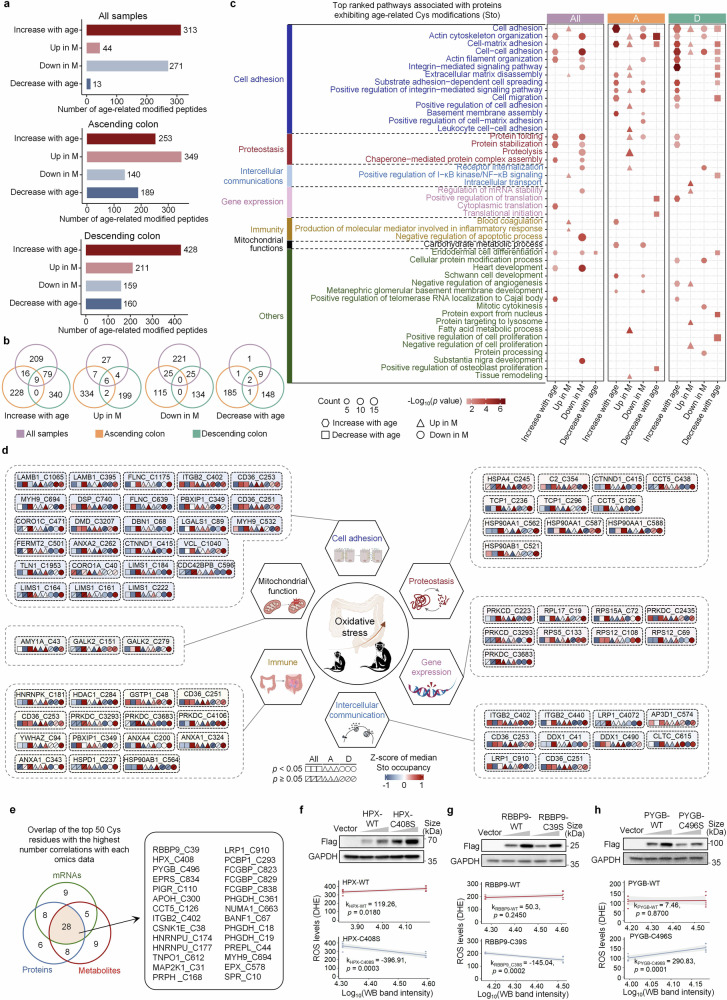
Location-resolved changes in Cys oxidation with age. **a** Statistics of peptides with Sto levels showing significant changes with age, with or without consideration of location effects. Peptides detected in at least three samples from each young, middle-aged, and old group were analyzed via one-way ANOVA (*p* < 0.05). Peptides detected in at least half of the samples in one or more groups but not detected in others were also classified as differentially oxidized. Up in M means highest abundance in the middle-age, and Down in M means lowest abundance in the middle-age. **b** Venn diagram displaying the number of overlapping modified peptides with or without considering location effects for each trend. **c** Bubble chart displaying the enriched Gene Ontology Biological Process (GOBP) using proteins with significantly changed Sto levels in all samples without considering location effects (All), in the ascending colon (A) and in the descending colon (D). The union of the top 5 enriched processes from each group is shown. These processes are divided into seven categories, as shown on the left. Different shapes represent different trends. The shape size indicates the protein number, and the color indicates −Log_10_ (*p* value). **d** Diagram showing representative Cys residues from six process categories. The color represents *z*-scored Sto levels across age groups. Symbols denote analysis without location effects (square), in the ascending colon (triangle), and in the descending colon (circle); slashes indicate nonsignificant age-related changes (one-way ANOVA). Elements were created via BioRender. **e** Venn diagram showing overlap of the top 50 Cys residues exhibiting the highest number of correlations with each omics dataset, including the transcriptome, proteome, and metabolome. **f**–**h** ROS level comparisons between wild-type (WT) and mutated (Cys to Ser mutation) genes, including *HPX* (**f**), *RBBP* (**g**), and *PYGB* (**h**), in response to 200 μM H_2_O_2_ treatment. “k” represents the slope of the line calculated via linear regression. A positive slope indicates increased ROS production; a negative slope indicates inhibition

Apart from those Cys residues with Sto changes spanning from Y1 to O2 or Y2 to O1, there are numerous Cys sites where the Sto levels change significantly with age, albeit not spanning two regions. Therefore, we comprehensively screened Cys residues with significantly altered Sto levels across age groups. Our analysis revealed a number of Cys residues whose Sto levels changed in different patterns, including increasing with age, decreasing with age, or exhibiting significant elevation or reduction in middle-aged individuals (Fig. 4a and Supplementary Table 4a). Notably, the overlap rate of various types of changes in Cys sites screened in the ascending colon and descending colon was remarkably low (Fig. 4b and Supplementary Table 4b), indicating disparities in the mechanisms involved in the response to oxidative stress between the two colon segments. Moreover, the number of Cys residues exhibiting age-related increases in Sto levels in the descending colon was markedly greater than that in the ascending colon, which is consistent with the above findings (Fig. 3i, m), suggesting a greater chance of oxidative stress in the descending colon. Pathway enrichment analysis revealed that most proteins were involved in the regulation of cell adhesion, proteostasis, gene expression, intercellular communication, immunity, and mitochondrial functions (Fig. 4c, d and Supplementary Table 4c, d). Although there was minimal overlap of Cys residues with significantly age-related Sto levels between the ascending and descending colons (Fig. 4b), the majority of common sites on the proteins within these pathways exhibited similar changes in Sto levels with age in both colon segments (Fig. 4d and Supplementary Table 4d).

Next, we are curious whether these oxidation modifications are merely a result of oxidative stress or if they also confer new functions to these proteins. We hypothesize that if the Sto levels of certain Cys residues exhibit more significant correlations with the abundances of other biomolecules, changes in the oxidation levels at those sites may have greater impacts on the cells and better reflect the effects of oxidative stress on cells. By using the Cys residues with age-related Sto changes, correlation analyses between the Sto levels of these Cys residues and the levels of various other molecules, including mRNAs, proteins, and metabolites, were conducted.^32^ Overlapping analysis of the top 50 Cys residues with the highest number of correlations with each omics dataset revealed 28 common residues (Fig. 4e and Supplementary Table 5a–i). To test whether Cys oxidation confers new functions to these proteins, we employed cell lines with site mutations of Cys to serine (Ser) on a specific gene to mimic the unoxidized state. By measuring intracellular reactive oxygen species (ROS), we compared the response to H_2_O_2_ treatment between mutated and non-mutated cell lines. We employed H_2_O_2_ treatment to make the comparison more pronounced. *HPX* is a protective gene that alleviates heme-induced oxidative stress.^33^ We found that mutating Cys to Ser in HPX-C408 significantly increased its antioxidative stress capabilities and reduced its cellular ROS levels following H_2_O_2_ treatment (Fig. 4f and Supplementary Table 5j), highlighting the importance of the unmodified C408 residue in combating oxidative stress. A similar result was observed when RBBP9-C39 was mutated to Ser, although the level of wild-type RBBP9 itself had no effect on ROS production (Fig. 4g and Supplementary Table 5j). In contrast, the PYGB-C496S mutation promoted an increase in ROS after H_2_O_2_ treatment (Fig. 4h and Supplementary Table 5j), indicating that the age-related increase in PYGB-C496 oxidation may play a role in combating oxidative stress.

Overall, we found that Cys oxidation occupancy exhibited a bimodal distribution, with some sites showing age-related shifts. In addition, we demonstrated that Cys oxidation not only indicates oxidative stress but also imparts new functions to these proteins, enabling them to either combat or promote oxidative stress.

### Exploring the metabolic regulators of oxidative stress

**Fig. 5:**
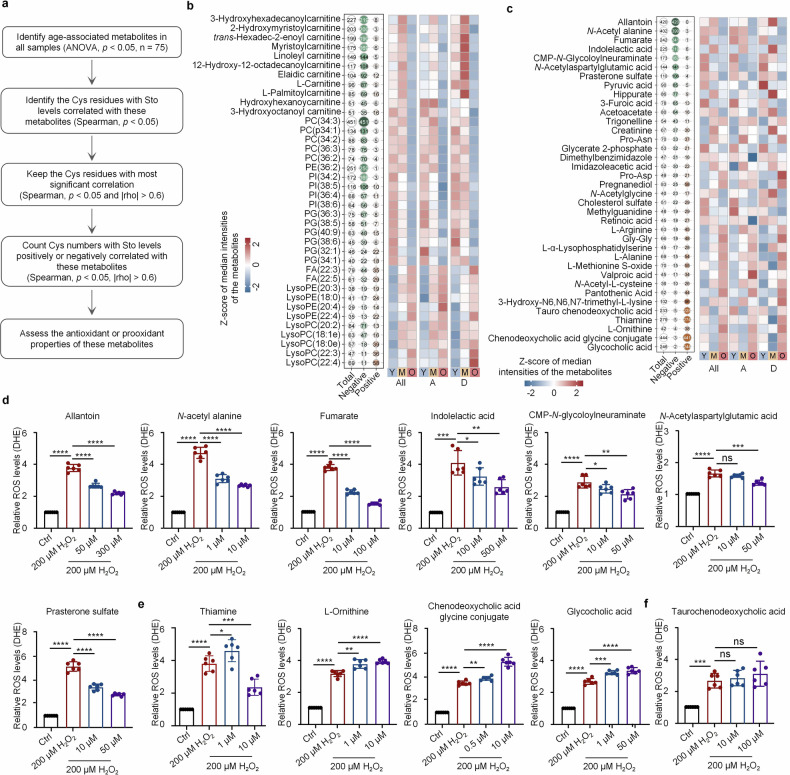
Metabolic regulation of oxidative stress during gut aging. **a** Schematic illustrating the process of identifying metabolites influencing oxidative stress. **b**, **c** List of metabolites significantly correlated with Sto levels. Carnitine and its derivatives, unsaturated fatty acid metabolites (**b**), and other metabolites (**c**) are shown. Left: Bubble chart showing the number of Cys residues with Sto levels correlated with metabolites, both positively and negatively. Right: Heatmap displaying z-scored median metabolite abundances across groups. All all samples irrespective of location effects, A ascending colon, D descending colon, Y young, M middle-aged, O old. **d**–**f** Intracellular ROS levels in NCM460 cells detected after treatment with 200 μM H_2_O_2_ and different metabolites (*n* = 6 independent experiments, mean ± s.d.). ^*^p < 0.05, ^**^p < 0.01, ^***^p < 0.001, and ^****^p < 0.0001; ns indicates no significance

There is growing evidence emphasizing the importance of the metabolic regulation of oxidative stress.^34^ Some metabolic pathways, including glycolysis, the tricarboxylic acid (TCA) cycle, and oxidative phosphorylation, produce ROS as byproducts.^35^ Disruptions in metabolic homeostasis, such as excessive caloric intake and nutrient imbalances, can exacerbate oxidative stress.^36^ While the antioxidative stress roles of some metabolites have been clarified,^37^ the functions of a greater number of other metabolites remain unclear. Moreover, our understanding of how these metabolites interact with the Cys redoxome to contribute to gut aging is limited. To gain insight into the metabolic regulation of oxidative stress, we initially identified 75 age-related metabolites in the gut without considering location-specific effects (ANOVA, *p* < 0.05). Spearman’s correlation analysis between the levels of the 75 metabolites and the Sto levels of all the Cys residues revealed that the Cys residues were significantly correlated with each metabolite (*p* < 0.05, |rho| > 0.6). Next, we tallied the number of Cys residues with Sto levels positively and negatively correlated with the metabolite levels and determined their potential properties in the regulation of oxidative stress. We hypothesized that the greater number of negatively correlated Cys residues suggests their potential roles in suppressing oxidative stress, whereas the greater number of positively correlated residues suggests their likely roles in promoting oxidative stress (Fig. 5a and Supplementary Table 6a). As a result, we identified several acylcarnitines, which are vital intermediates in fatty acid β-oxidation and are negatively associated with Sto levels, suggesting that mitochondrial dysfunction in aged individuals leads to increased ROS levels and oxidative stress (Fig. 5b and Supplementary Table 6b, c). Phosphatidylcholine (PC), phosphatidylethanolamine (PE), and phosphatidylglycerol (PG) are recognized antioxidants and are present at reduced levels in aged individuals.^38^ Consistently, several of these species presented negative correlations with Sto levels (Fig. 5b). More importantly, we noted that certain metabolites, such as allantoin, *N*-acetyl alanine, fumarate, indolelactic acid, CMP-*N*-glycoloylneuraminate, *N*-acetylaspartylglutamic acid, and prasterone sulfate, were negatively correlated with the Sto levels of numerous Cys residues, with decreased levels in the guts of aged individuals (Fig. 5c and Supplementary Table 6b, d), and that some metabolites, including glycocholic acid, chenodeoxycholic acid glycine conjugate, taurochenodeoxycholic acid and thiamine, were positively correlated with the Sto levels of many Cys residues, with increased levels in the guts of aged individuals (Fig. 5c). We also conducted a separate correlation analysis between the levels of Sto and metabolites in the ascending and descending colons and found distinct results between the two locations. Some metabolites were positively correlated with Sto levels at various sites in one location but negatively correlated with the other. The modification sites exhibiting these opposing correlations were different. However, the reason for this difference requires further exploration (Supplementary Fig. 1a–c and Supplementary Table 6a, e).

To validate their roles in regulating oxidative stress, we measured the intracellular ROS levels in H_2_O_2_-treated NCM460 cells following treatment with these key metabolites. Consistently, treatment with metabolites exhibiting more negative correlations with the Sto levels of Cys residues resulted in significantly lower levels of ROS (Fig. 5d and Supplementary Table 6f). Conversely, treatment with metabolites displaying more positive correlations with the Sto levels of Cys residues promoted ROS production (Fig. 5e and Supplementary Table 6f), indicating the reliability of our data analysis strategy. Notably, thiamine plays a dual role in regulating ROS, promoting ROS at a low concentration but exerting antioxidant effects at a high concentration. Additionally, we observed one exceptional metabolite, taurochenodeoxycholic acid, which did not show the anticipated effects under H_2_O_2_ treatment in NCM460 cells (Fig. 5f and Supplementary Table 6f).

**Fig. 6:**
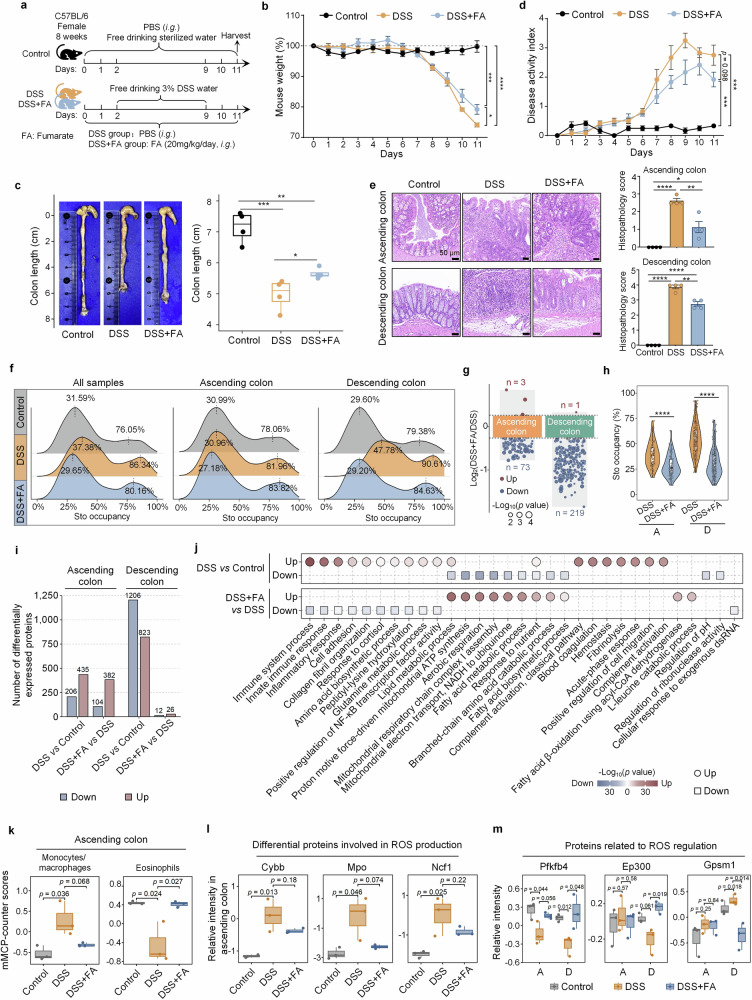
Fumarate alleviates oxidative stress in the mouse gut. **a** Flow chart of the experimental design. **b** Daily mouse body weight (Student’s *t-*test, mean ± s.e.m.). ^*^p < 0.05, ^***^p < 0.001, and ^****^p < 0.0001. **c** Representative images of mouse colon length and statistical analysis (Student’s *t-*test, mean ± quartiles). ^*^p < 0.05, ^**^p < 0.01, and ^***^p < 0.001. **d** Disease activity index determined by weight loss, stool consistency, and bleeding (Student’s *t-*test, mean ± s.e.m.). ^***^p < 0.001. **e** H&E images of the ascending and descending colons. Scale bars, 50 μm. Bar plots showing the statistical results of the histopathology score (0 = none; 1 = very mild; 2 = mild; 3 = moderate; 4 = severe) (Student’s *t-*test, mean ± s.e.m.). ^*^p < 0.05, ^**^p < 0.01, and ^****^p < 0.0001. **f** Density plots showing the distribution of Sto occupancy across different groups, with or without consideration of location effects. **g** Volcano plots showing sites with differential oxidation modifications in the ascending and descending colon of the DSS and DSS + FA groups (Student’s *t*-test, *p* < 0.05; Log_2_(DSS + FA/DSS) > 0.263 (red) or < −0.263 (blue)). The circle size indicates −Log_10_(*p* value). **h** Box plots showing the sites in the ascending and descending colons where Sto occupancy significantly decreased after fumarate intervention in the DSS and DSS + FA groups (Student’s *t-*test). ^****^p < 0.0001. **i** Bar plots showing the number of differentially expressed proteins between the control and DSS groups or between the DSS and DSS + FA groups in the ascending and descending colon. **j** Top ten GOBP pathways identified via DAVID in the ascending colon. **k** Boxplots illustrating the mMCP-counter scores in the ascending colon among the control, DSS, and DSS + FA groups (Student’s *t-*test). **l** Boxplots depicting the relative protein intensity in the ascending colon among the control, DSS, and DSS + FA groups (Student’s *t-*test). **m** Boxplots showing the relative protein intensity in the ascending and descending colons among the control, DSS, and DSS + FA groups (Student’s *t-*test). The samples are categorized as “All” (without location effects), “A” (ascending colon), and “D” (descending colon). DSS dextran sulfate sodium, FA fumarate

To further explore the impact of metabolites on oxidative stress, we used fumarate as an example because of its notable antioxidative properties (Fig. 5d). Dextran sulfate sodium (DSS) can disrupt the intestinal barrier, leading to excessive ROS production and oxidative stress. This exacerbates inflammation, damages tissues, and impairs mitochondrial and antioxidant functions. We evaluated the ability of fumarate to combat oxidative stress in a DSS-induced colitis mouse model (Fig. 6a). Our findings revealed that fumarate protected against weight loss (Fig. 6b) and gut length shortening (Fig. 6c) while reducing colitis severity (Fig. 6d) in both the ascending and descending colons (Fig. 6e). Consistent with these findings, redoxomic analysis revealed a more pronounced recovery of Cys oxidation in the guts of DSS- and fumarate-treated mice, particularly in the descending colon, than in the guts of DSS-treated mice (Fig. 6f and Supplementary Table 7a). Moreover, fumarate downregulated the oxidation of hundreds of Cys residues (Fig. 6g and Supplementary Table 7b), suggesting that it inhibits DSS-induced oxidative stress. The number of Cys sites with decreased Sto was greater in the descending colon (Fig. 6h and Supplementary Table 7b). Further stoichiometry analysis of individual Cys residues revealed that DSS treatment had a greater effect on the descending colon, a trend consistent with the more obvious stoichiometric changes observed in the descending colon during aging (Figs. 3h, i and 4a). After fumarate treatment, the Sto stoichiometries in both the ascending and descending colons nearly returned to the same level (Fig. 6h).

To understand how fumarate combats oxidative stress, we conducted a proteomics analysis. We found that fewer differential proteins were identified in the descending colon after fumarate treatment, which may be attributed to the shorter recovery period of 2 days (Fig. 6i and Supplementary Table 7c, d). Enrichment analysis of differentially expressed proteins in the ascending colon revealed that fumarate significantly inhibited immune and inflammatory response processes. Moreover, proteins involved in mitochondrial respiration were upregulated, helping mitigate the increase in ROS production caused by DSS-induced damage to the mitochondrial electron transport chain (Fig. 6j and Supplementary Table 7e). We also used a murine microenvironment cell population counter (mMCP-counter)^39^ to estimate the abundance of immune and stromal cells in the ascending colon. DSS treatment significantly increased the mMCP-counter score for monocytes/macrophages, which are known to promote colitis in mice,^40,41^ whereas the score decreased following fumarate treatment (Fig. 6k and Supplementary Table 7f). In contrast, eosinophils, which play a protective role in maintaining intestinal integrity,^42^ exhibited different response patterns than monocytes/macrophages did after DSS and fumarate treatments (Fig. 6k). Additionally, we observed that DSS treatment upregulated three proteins associated with superoxide generation and oxidative stress, Cybb,^43^ Mpo,^44^ and Ncf1,^45^ whereas fumarate treatment reversed these changes (Fig. 6l and Supplementary Table 7g). Immunohistochemical analysis revealed that the ascending colon, which experienced less damage, recovered more quickly than did the descending colon, which showed slower recovery (Fig. 6e). We identified several differentially expressed proteins in the descending colon after fumarate treatment (Fig. 6i). Although their number was limited, these proteins may be important for early intestinal recovery. Among them, Pfkfb4 and Ep300 are known to have antioxidative stress effects,^46,47^ whereas Gpsm1 has proinflammatory effects^48^ (Fig. 6m and Supplementary Table 7h).

Overall, we clarify the comprehensive crosstalk between the redoxome and metabolome and identify a number of crucial metabolites that are endogenous regulators of oxidative stress in the gut.

### Effects of CR intervention on redox signaling in the gut

**Fig. 7:**
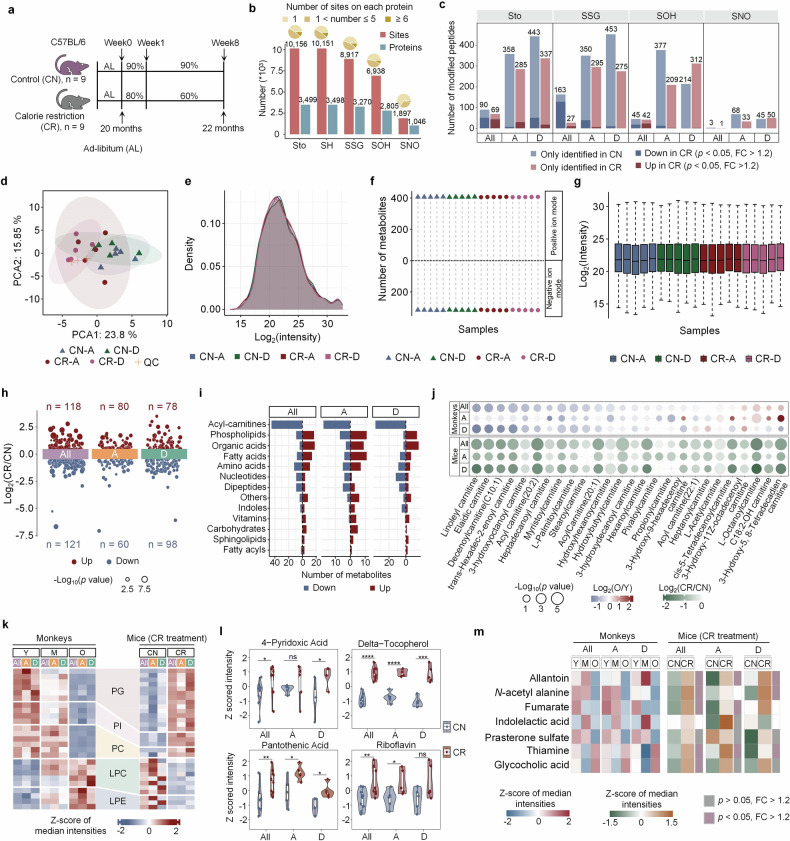
CR partially reverses the metabolic changes in the guts of aged mice. **a** Experimental design of the CR test in aged mice. **b** Bar graph depicting the number of modification sites and proteins and pie charts showing the distribution of quantified Cys numbers per protein. **c** Statistics of significantly altered peptides between the CR and CN groups (Student’s *t*-test). Light blue/red indicates peptides detected in at least half of the CN/CR samples, whereas dark blue/red indicates peptides detected in at least three samples from both groups. **d** Principal component analysis (PCA) of metabolome data from the colon tissues of 20 mice, with CN-A/D (control) and CR-A/D (CR) representing ascending/descending colon samples. **e** Density plot showing metabolite intensities across the four groups. **f** Number of metabolites detected in each sample under both ion modes. **g** Boxplots of log2-transformed normalized intensity of identified metabolites (*n* = 724) in 20 samples (median ± quartiles), with 5 samples for each group. **h** Volcano plot showing differentially abundant metabolites in All, A, and D samples between the CN and CR groups (Student’s *t*-test, *p* < 0.05; Log_2_(CR/CN) > 0.263 (red) or < −0.263 (blue)). The circle size indicates −Log_10_(*p* value). **i** Bar graphs indicating differentially abundant metabolite numbers in each category in All, A and D samples (Student’s *t*-test, *p* < 0.05; Log_2_(CR/CN) > 0.263 (red) or < −0.263 (blue)). **j** Comparison of acylcarnitine changes post-CR in mice vs aging monkeys. The circle size indicates −Log_10_(*p* value). **k** Heatmap showing reversed phospholipid levels in post-CR mice compared with those in aging monkeys. **l** Violin plots of four vitamins in All, A, and D samples post-CR (mean ± quartiles). Student’s *t-*test, ^*^p < 0.05, ^**^p < 0.01, ^***^p < 0.001, and ^****^p < 0.0001. “ns” indicates no significance. **m** Heatmaps of validated metabolite levels post-CR in mice vs aging monkeys. Purple and gray indicate significant and insignificant changes, respectively, post-CR (Student’s *t*-test). The samples are categorized as “All” (without location effects), “A” (ascending colon), and “D” (descending colon). CN control group, CR calorie restriction group

CR refers to the intentional reduction in calorie intake without malnutrition.^49,50^ It potentially affects various aspects of the gut, including microbiota composition, gut barrier integrity, inflammation levels, autophagy, and hormone regulation.^51^ While fasting for 24 h increases oxidized Cys residues in *Drosophila melanogaster*,^31^ other studies suggest that CR reduces ROS generation and oxidative stress, potentially extending lifespan,^52–54^ indicating that the effects of CR on intestinal oxidative stress and the Cys redoxome remain unclear. To clarify this, we applied a 2-month CR intervention to 20-month-old mice^55,56^ (Fig. 7a and Supplementary Tables 8a, 9a). Profiling of the Cys redoxome in the ascending and descending colons revealed thousands of Cys redox modifications (Fig. 7b and Supplementary Table 8b, c). Our results revealed a decrease in the number of Cys residues with differential redox modifications when location differences were not considered (fold change > 1.2 and Student’s *t-*test, *p* < 0.05) (Fig. 7c and Supplementary Table 8d), indicating distinct effects of CR on different colon regions. Similar to the findings in monkeys, we observed two distinct Sto occupancy peaks (Supplementary Fig. 2a and Supplementary Table 8e, f). The occupancy of many Cys residues shifted across the boundary separating these peaks, with more residues showing reduced Sto levels without considering location influences (Supplementary Fig. 2b–d and Supplementary Table 8e, f) or specifically in the descending colon (Supplementary Fig. 2e–g and Supplementary Table 8e, f) after CR intervention. In contrast, many fewer Cys residues in the ascending colon displayed reduced Sto occupancy following CR (Supplementary Fig. 2h–j and Supplementary Table 8e, f). Pathway analysis revealed that the Sto levels of proteins involved in cell adhesion decreased in both the ascending and descending colon following CR intervention. Proteins associated with cell growth-related pathways, such as cell cycle, glucose metabolism, and cell division, also exhibited reduced Sto levels, irrespective of location effects. However, these trends were not statistically significant when location effects were considered, probably due to the limited sample size (Supplementary Fig. 3a and Supplementary Table 8g). Interestingly, proteins involved in lipid metabolic processes displayed reduced Sto levels in the descending colon but increased Sto levels in the ascending colon. Additionally, proteins in certain pathways, such as protein transport, exhibited significant changes in Sto levels exclusively in either the ascending or descending colon (Supplementary Fig. 3a and Supplementary Table 8g). Similar pathway analyses identified proteome-wide changes in SSG, SOH, or SNO signaling after CR intervention (Supplementary Fig. 3b–d and Supplementary Table 8g).

Given the close relationships between metabolism and redox modifications, we explored how CR influences the metabolism of gut tissues and whether these metabolic changes are linked to alterations in redox modifications. Metabolomic analysis of gut tissues identified many metabolites affected by CR intervention (Fig. 7d–h and Supplementary Table 9a, b). Our previous findings indicated that many acyl carnitines are negatively correlated with Cys oxidation and decrease with age in monkeys (Fig. 5b). Statistical analysis revealed reduced acylcarnitine levels in the CR-treated group (Fig. 7i, j and Supplementary Table 9a, c, and d), suggesting that CR further suppressed mitochondrial fatty acid β-oxidation (FAO). In contrast, some PC, PG, and PE species (Fig. 7k and Supplementary Table 9e), as well as vitamins (Fig. 7l) were significantly reversed or upregulated, potentially playing an important role in combating intestinal oxidative stress. More importantly, we found that allantoin, *N*-acetyl alanine, fumarate, indolelactic acid, and prasterone sulfate, which have been demonstrated to suppress ROS production in this work (Fig. 5d), were reversed by CR. In contrast, glycocholic acid and thiamine, which promote ROS production, were further upregulated following CR intervention (Fig. 7m and Supplementary Table 9f). Although the changes in these metabolites are inconsistent in their ability to suppress oxidative stress, the recovery of many metabolites and antioxidants by CR may play a key role in reducing oxidative stress in the gut.

## Discussion

In summary, this study yields several significant advancements across several fronts. First, we improved the methodology for Cys redoxome profiling, enhancing both efficiency and accuracy while reducing sample input and costs. Second, we determined the stoichiometries of various Cys redox modifications and analyzed their dynamics during gut aging. Our pioneering investigation of redoxomics in the normal tissues of non-human primates yielded findings that closely mirror those that happened in humans. Third, we developed a reliable crosstalk analysis strategy, identifying numerous metabolites as key endogenous regulators of oxidative stress. Additionally, we showed that CR reprograms cellular metabolism, potentially affecting intestinal oxidative stress and gut aging.

Enriching low-abundance modified peptides is crucial for redoxomics. Biotin-based strategies offer efficient enrichment but pose elution challenges.^28,29,57^ Light-sensitive biotin probes have been developed to ease elution but add procedural complexity.^30,31^ TMT labeling is widely used for peptide quantification but requires substantial sample input and isobaric reagents, increasing total costs.^58–61^ Recently, a new strategy using cysteine-reactive phosphate tags (CPTs) has increased coverage and reproducibility for Cys redoxome quantification.^24^ Our method combines biotin for peptide enrichment and TMT for quantification, requiring only 10 μg of peptide per Cys modification, minimizing TMT use. Using hexafluoroisopropanol,^62^ the strong interaction between biotin and streptavidin can be rapidly and efficiently disrupted. Notably, despite reducing protein usage for isobaric labeling by more than 10-fold, our method maintains high redoxome throughput and simultaneously quantifies up to five distinct Cys redox states for each sample. Future adaptations involving efficient reduction and biotin labeling of modifications like SO_2_H and SO_3_H could extend this capability with advanced multiplexing TMT reagents.^63^ However, we must acknowledge that while TMT labeling is a powerful quantitative technique, the coelution of peptides and isotope contamination from other channels may impact quantification accuracy.

Oxidative stress results from an imbalance between free radical production (e.g., ROS) and antioxidant defense mechanisms involving glutathione (GSH), vitamins, flavonoids, polyphenols, and melatonin.^64^ When antioxidants are insufficient to neutralize these radicals, oxidative stress accelerates organ aging and increases disease risk.^65^ In addition to known antioxidants, metabolites such as fumarate can reduce oxidative stress, inhibiting the p38 MAPK-dependent NF-κB pathway and activating the Nrf2 pathway to bolster cellular defenses.^66,67^ However, fumarate can also react with GSH to form succinate GSH, paradoxically increasing ROS and oxidative stress, suggesting its dual regulatory role.^68,69^ Our research revealed that fumarate reduced DSS-induced intestinal injury and oxidative stress while lowering Cys oxidation levels. Furthermore, publicly available data indicate age-associated decreases in plasma fumarate levels.^70,71^ Similar reductions also occur in the brain with age.^72^ These findings suggest the potential systemic protective role of fumarate against oxidative stress. Although Cys residues are not classic antioxidants, the thiol groups on certain proteins are highly reactive in scavenging free radicals.^73^ Cys modifications serve as molecular switches in response to oxidative stress, which may activate or deactivate metabolic enzymes,^74,75^ leading to metabolic fluctuations, which may in turn affect oxidative stress.

CR plays a crucial role in reducing oxidative stress in the gut.^52–54,76^ It lowers ROS production and enhances antioxidant defense through pathways involving redox-sensitive proteins regulated by Nrf2.^77^ Moreover, CR affects the composition of the gut microbiota^78^ and shapes the intestinal redox environment through metabolites such as well-known short-chain fatty acids and secondary bile acids. It also affects energy-sensing pathways such as the AMPK and mTOR pathways, enhancing fatty acid oxidation and mitochondrial biogenesis and thus maintaining cellular homeostasis.^79,80^ In our study, we not only examined the reversal of certain metabolites that combat oxidative stress (Fig. 7m), but also assessed changes in gene expression following CR intervention. We found that some pro-aging genes,^32^ such as *EPX*, *SCIN*, *STX1A*, and *ATP1A3*, exhibited a more pronounced reversal in the ascending colon (Supplementary Fig. 4 and Supplementary Table 8i). In contrast, the levels of pro-aging genes,^32^ such as *LIPE*, *SERPINC1*, and *NFU1*, continued to increase in the ascending colon, whereas CR reversed their expression in the descending colon (Supplementary Fig. 4 and Supplementary Table 8i). Given the complexity of the effects of CR on oxidative stress and gut aging, the function and mechanisms of the antiaging effects of CR require further evidence.

Overall, this work describes a low-input redoxomics technique, decodes the complexities of redoxomics during the gut aging of non-human primates, and identifies key metabolic regulators of oxidative stress and redox signaling.

## Methods

### Animal information and ethics approval

*Macaca fascicularis* were housed in a uniformly controlled laboratory in Canton and maintained at approximately 25 °C with a 12-h light and 12-h dark cycle. All the animals had no previous clinical or experimental background that could affect their natural aging process or increase their vulnerability to illnesses. Tissues from the colons of 4 young (females, 3–4 years old), 4 middle-aged (females, 9–10 years old), and 4 old (females, 15–16 years old) *Macaca fascicularis* were excised and immediately frozen in liquid nitrogen for preservation. This study adhered to the ethical guidelines for the treatment of non-human primates and was approved by the Institutional Animal Care and Use Committee at Yuanxi Biotech Inc. in Guangzhou (approval number: YXSW-2016-01).

C57BL/6J mice were purchased from GemPharmatech Co., Ltd. (Jiangsu, China). All the animals were kept in a specific pathogen-free (SPF) animal facility with a 12 h photoperiod. Before the experiment, the mice were cohoused, provided with a normal chow diet, and housed with ad libitum access to water. For the calorie restriction (CR) experiments, the mice were randomly grouped. The utilization and welfare of the mice were authorized by the Animal Experiment Ethics Committee of the State Key Laboratory of Biotherapy at Sichuan University (approval number: 20230307027).

### Procedures for redoxomics

Frozen colon tissues from monkeys or mice were lysed with HEPES buffer (10 mM EDTA, 250 mM HEPES, 0.1 mM neocuproine, pH 7.5, 0.5% SDS, and 1% protease inhibitor cocktail) and homogenized twice via gentleMACS^TM^ following the procedure of protein_01.01. The crude lysate was then sonicated in an ice water bath for 5 min, followed by centrifugation at 20,000×*g* for 30 min at 4 °C. The supernatant was obtained, and the concentration was determined via a bicinchoninic acid (BCA) assay (23227, Thermo Fisher Scientific).

To label the free Cys residues (SH) with a biotin reagent, the protein was diluted to 1 μg/μL in reaction buffer (5 mM EDTA, 50 mM Tris HCl, pH 8.3). Then, the mixture was incubated with 0.8 mM iodoacetyl-PEG2-biotin (21334, Thermo Fisher Scientific) in the dark for 90 min at room temperature. Afterward, the mixture was precipitated with methanol/water/chloroform. Following precipitation, 50 μL of resuspension buffer (250 mM HEPES, pH 7.7, 0.1% SDS, and 8 M urea) and 50 μL of reaction buffer were added. The protein concentration was quantified again via the BCA assay. Next, 5 mM Tris(2-carboxyethyl) phosphine (TCEP, C4706, Sigma-Aldrich) was added, and the mixture was incubated at 56 °C for 1 h. Subsequently, 10 mM iodoacetamide (IAM, I1149, Sigma-Aldrich) was added, and the mixture was incubated in the dark for 30 min at room temperature to allow for alkylation. After the reaction, 50 μg of protein was isolated, precipitated with methanol/water/chloroform, and digested with sequencing-grade modified trypsin (V5117, Promega).

To label Cys total oxidation (Sto) with a biotin reagent, the protein sample was mixed with 10 mM IAM and incubated to block free thiol groups. The protein was then precipitated to remove excess IAM. Next, the protein was redissolved in 50 μL of resuspension buffer and 50 μL of reaction buffer, followed by reduction with 5 mM TCEP. To remove excess TCEP, the protein was precipitated again and subsequently redissolved in 50 μL of resuspension buffer and 50 μL of reaction buffer. The concentration was reassessed via the BCA assay. Iodoacetyl-PEG2-biotin (1.2 mM) was added, and the mixture was incubated for 90 min. Finally, 50 μg of protein was precipitated and then digested with trypsin for 16 h.

To label Cys residues with SOH or SNO modifications with a biotin reagent, the protein was diluted to 1 μg/μL with 100 mM TEAB. A total of 100 μg of protein was subsequently combined with 10 mM IAM to block free thiol groups. The protein was precipitated via methanol/water/chloroform and then resuspended in 50 μL of HENS buffer (1 mM EDTA, 250 mM HEPES, pH 7.7, 0.1 mM neocuproine, and 1% SDS) and 50 μL of reaction buffer. Next, 20 mM sodium arsenite (for SOH) or sodium ascorbate (for SNO) and 0.4 mM iodoacetyl-PEG2-biotin were added and incubated. To remove excess reagents, precipitation was performed. The protein was redissolved in 50 μL of resuspension buffer and 50 μL of reaction buffer, and its concentration was reassessed using the BCA assay. The protein was subsequently reduced by incubation with 5 mM TCEP, followed by alkylation with 10 mM IAM. Finally, 50 μg of protein was precipitated, and digestion was carried out with trypsin.

To label Cys residues with SSG modification with a biotin reagent, 100 μg of protein lysates were diluted to 1 μg/μL with 100 mM TEAB and treated with 10 mM IAM. After blocking, the protein was precipitated with methanol/water/chloroform and then redissolved in a resuspension buffer. Buffer exchange was performed using 0.5 mL Amicon Ultra 10 K filter units (Millipore, Burlington, MA, USA) with exchange buffer (25 mM HEPES, 1 M urea, pH 7.5). The protein concentration was quantified via the BCA method. The samples were diluted to a concentration of 1 μg/μL with exchange buffer. To reduce the SSG, the following components were added to the solution: 2.5 μg/μL glutaredoxin 1 M (Grx1M, C14S), 0.25 mM oxidized glutathione (GSSG, G8690, Solarbio), 4 U/mL glutathione reductase (GR, P2372S, Beyotime), and 1 mM reduced nicotinamide adenine dinucleotide phosphate (NADPH, ST360, Beyotime). The samples were maintained at 37 °C for 10 min to allow for incubation, quickly chilled on ice, and transferred to a 0.5 mL Amicon Ultra 10 K filter. Excess reagents were removed by washing with reaction buffer to a final volume of 100 μL, and the concentration was quantified using a BCA method. The sample was then reacted with 0.4 mM iodoacetyl-PEG2-biotin and precipitated with methanol/water/chloroform. After precipitation, 50 μL of resuspension buffer and 50 μL of reaction buffer were added. The protein concentration was reassessed via the BCA assay. The sample was then reduced with 5 mM TCEP and alkylated with 10 mM IAM. Finally, 50 μg of protein was precipitated and digested with trypsin.

To label all the Cys residues with a biotin reagent, equal protein amounts from each sample were taken and combined to create a mixed sample. The concentration was assessed via the BCA method. The mixed sample was diluted to 1 μg/μL with 100 mM TEAB, reduced with 5 mM TCEP, and precipitated to remove excess TCEP. The pellet was dissolved in 50 μL of resuspension buffer and 50 μL of reaction buffer, and the protein concentration was again assessed via BCA. Next, alkylation was carried out with 2 mM iodoacetyl-PEG2-biotin. The protein was then precipitated with methanol/water/chloroform and subjected to digestion with trypsin.

### TMT labeling for redoxomics

Redoxomic analysis of *Macaca fascicularis* and mouse colon tissue samples was performed via a tandem mass tag (TMT)-based strategy. Each batch of TMT-labeled samples included a shared reference sample, which was biotin labeled with Cys residues (Sall) and labeled with TMT-127. The other five channels of the TMT-6plex reagent (Thermo Scientific) were assigned to label five different Cys redox states of the same sample: TMT-126 for Sto, TMT-128 for SH, TMT-129 for SNO, TMT-130 for SOH, and TMT-131 for SSG. For each TMT channel, 10 μg of peptide was isobarically labeled. The six channels, with a total of 60 μg of peptide, were combined and then dried via a SpeedVac. The dried peptides were desalted via a C18 solid-phase extraction (SPE) column (10 mg, Waters). Notably, in the redoxomic analysis of colon tissues from DSS-induced mice, the reference sample (Sall) was labeled with TMT-127. The free SH groups in the ascending colon samples were labeled with TMT-126, the Sto groups in the ascending colon samples were labeled with TMT-129, the free SH groups in the descending colon samples were labeled with TMT-130, and the Sto groups in the descending colon samples were labeled with TMT-131.

### Enrichment of biotinylated peptides using streptavidin magnetic beads

Labeled peptides (60 μg) were dissolved in 60 μL of 50 mM TEAB. Among these, 4 μg was desalted and subjected to LC-MS/MS analysis. This measurement was intended to determine the protein amounts in each channel, which would be utilized for normalizing the redox modification ratios. The remaining biotin-labeled peptides were enriched via streptavidin magnetic beads (HY-K0208, MCE). The mixture was then incubated at 4 °C for 6 h. The streptavidin magnetic beads were washed sequentially once with 1 mL of PBST (150 mM sodium chloride, 20 mM potassium phosphate, 0.5% Tween-20, pH 7.2), 500 μL of 1 M NaCl, 2 mL of PBST, 500 μL of PBS, and 500 μL of ddH_2_O. Finally, 100 μL of 1,1,1,3,3,3-hexafluoro-2-propanol (HFIP, 105228, Sigma-Aldrich) was used to elute the biotinylated peptides at 59 °C for 5 min, and this elution step was repeated 3 times. All eluates were combined and concentrated to dryness. The eluted samples were then desalted via a C18 ZipTip and analyzed via LC-MS/MS.

### Sample preparation and TMT labeling for proteomics

Protein extraction, reduction, alkylation, and enzymatic digestion of colon tissues were carried out as previously described.^32^ A proteomic analysis of mouse colon tissues employed a TMT-based approach, where every batch of TMT-labeled samples included a shared reference sample. For calorie-restricted mice, the reference sample was tagged with TMT-127N. Twenty samples were tagged using the other ten channels of the TMT-11plex reagents (Thermo Scientific). The labeling process adhered to the instructions outlined in the TMT kit, with each sample containing 70 μg of peptides being labeled. After quenching with 5% hydroxylamine, the samples were combined and used for proteomics analysis. For the fumarate treatment of DSS-treated mice, the reference sample was tagged with TMT-128C. Eighteen samples were tagged via the other nine channels of the TMT-10plex reagents (Thermo Scientific). The labeling procedure followed the TMT kit instructions and used 10 μg of peptides per sample. The labeled peptides were redissolved in 0.1% TFA and desalted with C18 SPE columns (10 mg, Waters), followed by fractionation with high-performance liquid chromatography (RP-HPLC; LC2030 plus; Shimadzu). Briefly, a total of 50 μg of TMT-labeled peptides were fractionated via a Poroshell HPH C18 column (250 × 4.6 mm, OD 4 μm, Agilent) at a flow rate of 1 mL/min. The fractionation process involved the use of solvent A (98% water, 2% acetonitrile, pH 10) and a gradient increase in solvent B (10% water, 90% acetonitrile, pH 10). The 120-min LC gradient was set as follows: 0–2% B in 2 min, 2–35% B in 58 min, 35–55% B in 35 min, 55–90% B in 21 min, and 90–2% B in 4 min. The initially obtained 120 fractions were combined into 20 fractions, which were subsequently dried and desalted via Zip-tip columns.

### Procedures for untargeted metabolomics

Approximately 30 mg of mouse colon tissue and four steel beads were placed in 500 μL of precooled 80% methanol. The tissue was homogenized via Precellys Evolution Touch at 6800 rpm for 10 s, followed by a 30 s pause, and this process was repeated three times. Afterward, 500 μL of 80% methanol was introduced into the mixture and vortexed at 1500 rpm for 30 s. The lysate was then incubated at −80 °C for 30 min, ultrasonicated in an ice water bath for 10 min, and centrifuged at 13,000 rpm for 20 min at 4 °C. Following centrifugation, 600 μL of the supernatant was obtained, and 500 μL of 80% methanol was added again. The mixture was vortexed and centrifuged, and the supernatants were combined. Additionally, 200 μL of extracted metabolites from each sample were pooled to serve as the QC for mass spectrometry (MS) analysis. The supernatants from all the samples were concentrated and evaporated under a vacuum. The protein precipitate was dried and redissolved in 0.1 M KOH, and the concentration was determined via BCA for subsequent data calibration.

### LC-MS/MS analysis

For the analysis of the enriched biotinylated peptides, an EASY-nanoLC 1000 system (Thermo Fisher Scientific) coupled with a Q Exactive Plus mass spectrometer (also from Thermo Fisher Scientific) was used to perform LC-MS/MS analysis. The desalted peptides were reconstituted in buffer A (98% water, 2% acetonitrile, 0.1% formic acid) and separated via a custom trap column (2.5 cm × 75 μm) filled with Spursil C18 particles (5 μm; DIKMA), followed by an analytical column (25 cm × 75 μm) filled with Reprosil-Pur C18-AQ particles (1.9 μm; Dr Maisch). The separation was accomplished with buffer A and a nonlinear increase in buffer B (80% acetonitrile, 0.1% formic acid) using a gradient ranging from 14% to 100% across 120 min with a flow rate of 330 nL/min. Data-dependent acquisition (DDA) was carried out using Xcalibur software. The MS1 full scan was set to a range from 350 *m*/*z* to 1600 *m*/*z*, with a resolution of 70,000, the automatic gain control (AGC) set at 3e^6^, and the maximum ion injection time (IT) at 20 ms. Fragmentation was performed on the 15 most abundant parent ions using a normalized collision energy (NCE) of 30%. For MS2 analysis, a resolution of 35,000 was applied, with an AGC target of 1e^5^ and a maximum IT of 100 ms. An isolation window of 0.6 *m*/*z* was applied, and precursors with unassigned charge states or charge states of *z* = 1, 6–8, or unassigned charge states were not considered for further analysis.

To analyze the unenriched peptides for protein quantification in each channel, LC-MS/MS analysis was performed via the same instrumentation setup. A 65-min gradient, ranging from 13% to 100% Buffer B, was used for the separation of peptides at a flow rate of 330 nL/min. DDA was conducted in positive ion mode, and the parameter settings for MS1 and MS2 were the same as those described above.

For proteomics, LC-MS/MS analysis was carried out via an EASY-nanoLC 1000 (Thermo Fisher Scientific) coupled to a Q Exactive Plus mass spectrometer (Thermo Fisher Scientific). The desalted peptides were reconstituted in Buffer A. The peptides were separated via a custom trap column (2.5 cm × 75 μm) filled with Spursil C18 particles (5 μm; DIKMA) and an analytical column (25 cm × 75 μm) filled with Reprosil-Pur C18-AQ particles (1.9 μm; Dr. Maisch). Peptides were eluted with a 90-min gradient from 13% to 100% Buffer B with a flow rate of 330 nL/min. DDA was conducted using Xcalibur software. The parameter settings for MS1 and MS2 were identical to those described above.

Hydrophilic metabolites extracted from mouse colon tissues were analyzed via an Ultimate 3000 UHPLC (Dionex) coupled with a Q Exactive mass spectrometer (Thermo, CA). The temperature of the column was maintained at 35 °C. The specific parameters for metabolite detection were consistent with those used in previous methods.^32^

### MS database searching

Spectrometric raw data files from redoxomics and proteomics were processed using MaxQuant (version 1.6.1.0). The omics data from *Macaca fascicularis* colon tissues were aligned against a combined *Macaca fascicularis* protein sequence database, which contained 76,242 protein sequences. For mice, the omics data were aligned to the Swiss-Prot mouse protein sequence database, updated on 07/2019, which consists of 17,019 sequences. TMT-6plex was employed as the secondary ion quantification method, with a “Min. reporter PIF” set to 0.75. The analysis parameters included a reporter mass tolerance of 0.002 Da and a peptide mass tolerance of 10 ppm. The false discovery rates (FDRs) for peptides and proteins were kept below 1%. Enzymatic digestion allowed up to 2 missed cleavages by trypsin. The peptides were required to consist of at least six amino acids, with a maximum molecular weight limit of 12,000 Da. Methionine oxidation and acetylation at the N-terminus were assigned as variable modifications. For proteomics analysis, Cys carbamidomethylation was specified as a fixed modification, whereas for redoxomics analysis, Cys carbamidomethylation was designated a dynamic modification, and a new dynamic modification, “redox-biotin” ( + 414.194 Da), was added.

### Redoxomics data processing

The proteomics data used for normalizing the redoxomics data were processed as follows. The proteinGroups.txt files were processed via R (version 4.3.0). Contaminants and decoy proteins were removed. The sum abundance in each TMT channel was scaled by the sum abundance of the common reference to obtain a total protein ratio. This ratio was then used to normalize the ratio of redox modifications.

The redox-biotinSites.txt file, containing the redox modification data, was processed as follows. Peptides marked with “+“ for reverse or potential contaminants, as well as those labeled with “REV_“ and “CON_“, were removed. The intensity of each peptide was normalized to the total protein ratio to obtain the normalized intensity. The normalized intensity of each biotinylated peptide of each sample was divided by the normalized intensity of the same peptide in the common reference sample. Furthermore, biotinylated cysteine residues derived from SH, Sto, SSG, SOH, and SNO, with probabilities greater than 0.75, were retained.

### Proteomic data processing

For proteomics analysis, the “peptides.txt” file was processed via R (version 4.3.0). Contaminants and decoy proteins were removed, and unique peptides were extracted. Only proteins with ≥ 2 unique peptides were retained. The signal intensities of distinct unique peptides corresponding to the same protein were summed to quantify the overall abundance of the protein. To reduce sample loading variations, the total protein intensities in each sample were normalized to the same level. Additionally, protein intensities were further normalized against the common reference in each TMT batch to eliminate batch effects. Finally, all batches of data were merged, zeros were replaced with “NA,” the data were transformed to a log2 scale, and proteins identified in fewer than 50% of the samples were excluded from continued analysis.

### Metabolomic data processing

Raw data from untargeted metabolomics were processed via TraceFinder (Thermo, CA) on the basis of a custom-built metabolite database. The identification parameters included a mass tolerance of 10 ppm for precursor ions and 15 ppm for fragment ions, along with a permissible retention time shift of up to 0.25 min for quantitation. Metabolites with CV values ≥ 0.3 across quality control replicates were excluded to ensure reliability. Metabolite abundance was normalized by dividing each value by the corresponding protein concentration in the sample. Metabolites detected in positive and negative ion modes were normalized separately, with each metabolite’s intensity divided by a correction factor (correction factor = total intensity of each sample/average total intensity of all samples). Finally, the metabolites detected in both modes were combined.

### Stoichiometry analysis

For each Cys residue, the stoichiometry of modifications such as SSG, SOH, SNO, or Sto was calculated by dividing the intensity of each modification by the sum of the intensities of Sto and SH. Cys residues quantified in at least three samples per group were retained, and the median occupancy of each modification on each Cys residue across different groups was used to generate the density distribution map.

### Motif analysis

Motif analysis was based on cysteine (Cys) residues located within ±10% of the Sto occupancy peak in both young and old individuals. Peptide sequences comprising 10 amino acids both before and after the identified modification site, totaling 21 amino acids, were selected for analysis. The analysis was conducted using the ‘ggseqlogo’ package in R (version 4.3.0).

### Bioinformatics and statistical analysis

The proteomic, transcriptomic, phosphoproteomic, and metabolite data of the *Macaca fascicularis* colon tissues were normally distributed. To analyze differences in metabolite levels across three age groups, one-way ANOVA was used.

Differentially expressed redox modifications were screened via the following criteria. First, if the modification sites were detected in at least 3 samples in each age group, the disparities among the three age groups were evaluated using one-way ANOVA. Additionally, modification sites detected in more than half of the biological samples in one or two age groups but not detected in other age groups were also considered differential sites. To determine the age-related trends of these modifications, the fold changes between different age groups were compared.

The redoxomic data from mouse colon tissues were log2 transformed to ensure consistency with a normal distribution. For modification sites detected in at least three samples in both the control and the experimental groups, the Student’s *t-*test was used to evaluate statistically significant changes (*p* value < 0.05 and fold change > 1.2). Additionally, modification sites detected in more than half of the samples in one group but not in the other group were also defined as significantly different sites.

Spearman’s correlation analysis was used to assess the correlation between the two sets of omics data. Proteins significantly correlated with Cys modifications were subjected to Gene Ontology biological process (GOBP) enrichment analysis via the DAVID database. Metabolites significantly correlated with Cys modifications were subjected to Kyoto Encyclopedia of Genes and Genomes (KEGG) enrichment analysis via MetaboAnalyst. Additionally, for enrichment analysis of mRNAs significantly correlated with Cys modifications, gene set enrichment analysis (GSEA) utilizing the hallmark gene set was carried out. All data analyses and graphical representations were carried out via R (version 4.3.0) and GraphPad Prism 9.0.

### Procedures for assessing the functions of Cys residues

Full-length cDNAs encoding human genes (*HPX, PYGB*, *RBBP9*) were obtained from Miaoling Biology. Genes with point mutations (*HPX*-C408S, *PYGB*-C496S, and *RBBP9*-C39S) were constructed via PCR-mediated mutagenesis with 2X Phanta Max Master Mix (P525-01, Vazyme). The cDNAs were subsequently cloned and inserted into pLV3-CMV-MCS-3×FLAG-CopGFP-Puro vectors.

HEK293T cells were cultured in Dulbecco’s modified Eagle’s medium, while NCM460 normal human colon mucosal epithelial cells were grown in the RPMI-1640 medium. Both media were supplemented with 10% fetal bovine serum (FBS) (FSP500, ExCell Bio) and 100 U/mL penicillin plus 100 μg/mL streptomycin (10,099-141 C, Gibco). The wild-type and mutant genes packaged by lentiviruses were used to infect NCM460 cell lines. After 48 h, a portion of the cells was collected, and the expression of the wild-type and mutant proteins was detected via Western blotting.

The protein lysates were separated by 10% SDS‒PAGE and subsequently transferred onto a polyvinylidene fluoride (PVDF) membrane with a pore size of 0.22 μm (ISEQ00010, Millipore). After blocking, the samples were incubated overnight at 4 °C with primary antibodies (diluted 1:5000). HRP-conjugated secondary antibodies (diluted 1:10,000) were then incubated for 1 h at room temperature. Primary antibodies against Flag (66008-4-Ig, Proteintech) and GAPDH (60004-1-Ig, Proteintech) were utilized. The band intensity was evaluated via ImageJ.

NCM460 cells were harvested from a 10 cm culture dish and reseeded in a black 96-well plate. After 24 h, 200 μM H_2_O_2_ (Sigma-Aldrich, 88597) was added to the medium, and the mixture was incubated for 2 h. At the conclusion of the treatment period, the H_2_O_2_-containing medium was replaced with a fresh culture medium. After an additional 24 h, the medium was removed, and the cells were then rinsed thoroughly three times with Dulbecco’s phosphate-buffered saline (DPBS). Intracellular ROS levels were quantified via DHE (Dihydroethidium, Applygen, C1300-2). The cells were incubated with 100 μL of serum-free cell culture medium containing 10 μM DHE at 37 °C for 60 min. After incubation, the cells were washed with 1x PBS, and 150 μL of fresh DPBS was added to each well. Fluorescence measurements were then obtained via a microplate fluorescence reader (Bio-Tek) at excitation and emission wavelengths of 535 nm and 610 nm, respectively. Intracellular ROS production was normalized to the number of cells. The slope of ROS production was calculated relative to the expression levels of the wild-type or mutant proteins.

### ROS measurements in cells treated with metabolites

NCM460 cells were seeded in 96-well plates at a density of 4000 cells per well in RPMI-1640 culture medium (C11875500BT, Gibco) and incubated at 37 °C for 24 h. The cells were then divided into 3 groups: the control group, the H_2_O_2_-treated group, and the metabolite- and H_2_O_2_-treated groups. The metabolites were dissolved in DMSO (196055, MP Biomedicals), and the cells in the metabolite and H_2_O_2_-treated groups were cultured with serum-free medium containing metabolites for 6 h, whereas those in the control group and H_2_O_2_ group were cultured with serum-free medium. H_2_O_2_ (200 μM) was subsequently added to the H_2_O_2_-treated group and the metabolite- and H_2_O_2_-treated groups, and the cells were subsequently incubated for 2 h. Afterward, all groups of cells were incubated with fresh medium at 37 °C for an additional 24 h. Finally, the intracellular ROS levels were measured as described above. The following metabolites were evaluated: allantoin (05670, Sigma-Aldrich), *N*-acetyl-L-alanine (A4625, Sigma-Aldrich), fumaric acid (47910, Sigma-Aldrich), indolelactic acid (I157602, Aladdin), CMP-*N*-glycoloylneuraminate (BT-T15, Beijing Bette Renkang Biomedical Technology), *N*-acetylaspartylglutamic acid (S879667, Macklin), prasterone sulfate (D609795, Aladdin), taurochenodeoxycholic acid (T303865, Aladdin), thiamine (WKQ0788740, Sichuan Vicchi Biotechnology), l-ornithine (L413185, Aladdin), chenodeoxycholic acid glycine conjugate (G304255, Aladdin), and glycocholic acid (G131002, Aladdin).

### Fumarate treatment of DSS-induced colitis

C57BL/6 female mice (7-week-old) were fed for 7 days before the start of the treatment. After acclimatization, the mice were randomly divided into three groups. The DSS + FA group received a daily oral gavage of 20 mg/kg body weight fumarate dissolved in PBS, whereas the control (CON) and DSS groups were gavaged daily with an equivalent volume of PBS. During the colitis induction period (day 2 to day 9), the drinking water for the DSS + FA and DSS groups was replaced with a 3% DSS solution. The CON group received regular water. Each group consisted of four mice. Daily records of body weight and disease activity indices were obtained. At the end of the experiment, the mice were humanely euthanized via cervical dislocation, and colon tissues were promptly excised for length measurement to assess inflammation.

### Histopathology

Colon tissues were fixed in 4% paraformaldehyde, followed by embedding in paraffin. The samples were then sectioned into thin slices, each measuring 5 μm in thickness, and subsequently stained with hematoxylin‒eosin. The sections were imaged via an automated digital slide scanner (PANNORAMIC MIDI, 3DHISTECH) and evaluated with CaseViewer (RRID: SCR_017654). Histopathological scoring was performed by two independent observers in a double-blind manner. Previous specific scoring criteria were applied.^32^

### Calorie restriction of mice

One week prior to the calorie restriction experiment, 20-month-old mice were divided into two groups and housed in separate cages. The daily food consumption of the mice in each cage was recorded. During the initial week of the experiment, both the control normal (CN) group and the calorie restriction (CR) group were fed once a day. The CN group received 90% of their daily food intake, whereas the CR group received 80%. All the food provided was consumed by the animals. Beginning from the second week onward, the CN group continued receiving 90% of their daily food intake, whereas the CR group received 60% of their daily food intake. This regimen was maintained throughout the experiment, which lasted for two months. The mice were subsequently euthanized, and the ascending and descending colons were quickly separated and immediately frozen in liquid nitrogen for redoxomics, metabolomics, and proteomics analyses.

## Supplementary information

SUPPLEMENTAL MATERIALS

Supplementary Table 1

Supplementary Table 2

Supplementary Table 3

Supplementary Table 4

Supplementary Table 5

Supplementary Table 6

Supplementary Table 7

Supplementary Table 8

Supplementary Table 9

## Data Availability

Raw mass spectrometry-based redoxomic and proteomic data have been deposited in the ProteomeXchange Consortium (https://proteomecentral.proteomexchange.org) via the iProX partner repository^81,82^ with the dataset identifier PXD053818. The raw RNA sequencing data have been deposited in the Sequence Read Archive under accession numbers PRJNA1113585 and PRJNA999062.

## References

[1] Thursby, E. & Juge, N. Introduction to the human gut microbiota. *Biochem J.* **474**, 1823–1836 (2017). 28512250 10.1042/BCJ20160510 PMC5433529

[2] Warraich, U. E., Hussain, F. & Kayani, H. U. R. Aging—oxidative stress, antioxidants and computational modeling. *Heliyon* **6**, e04107 (2020). 32509998 10.1016/j.heliyon.2020.e04107 PMC7264715

[3] Wang, Y., Chen, Y., Zhang, X., Lu, Y. & Chen, H. New insights in intestinal oxidative stress damage and the health intervention effects of nutrients: a review. *J. Funct. Foods* **75**, 104248 (2020).

[4] Lobo, V., Patil, A., Phatak, A. & Chandra, N. Free radicals, antioxidants and functional foods: Impact on human health. *Pharmacogn. Rev.* **4**, 118–126 (2010). 22228951 10.4103/0973-7847.70902 PMC3249911

[5] Matthews, J. D. et al. Proteomic analysis of microbial induced redox-dependent intestinal signaling. *Redox Biol.* **20**, 526–532 (2019). 30508697 10.1016/j.redox.2018.11.011 PMC6275846

[6] Dennis, K. K., Go, Y. M. & Jones, D. P. Redox systems biology of nutrition and oxidative stress. *J. Nutr.* **149**, 553–565 (2019). 30949678 10.1093/jn/nxy306 PMC6461723

[7] Bhattacharyya, A., Chattopadhyay, R., Mitra, S. & Crowe, S. E. Oxidative stress: an essential factor in the pathogenesis of gastrointestinal mucosal diseases. *Physiol. Rev.* **94**, 329–354 (2014). 24692350 10.1152/physrev.00040.2012 PMC4044300

[8] Stavely, R. et al. Oxidative stress and neural dysfunction in gastrointestinal diseases: can stem cells offer a solution? *Stem Cells Transl. Med.* **12**, 801–810 (2023). 37774373 10.1093/stcltm/szad063 PMC10726411

[9] Mishra, B. & Jha, R. Oxidative stress in the poultry gut: potential challenges and interventions. *Front Vet. Sci.* **6**, 60 (2019). 30886854 10.3389/fvets.2019.00060 PMC6409315

[10] Li, L. et al. Oxidative stress, inflammation, gut dysbiosis: What can polyphenols do in inflammatory bowel disease? *Antioxidants* **12**, 967 (2023). 37107341 10.3390/antiox12040967 PMC10135842

[11] Alcock, L. J., Perkins, M. V. & Chalker, J. M. Chemical methods for mapping cysteine oxidation. *Chem. Soc. Rev.* **47**, 231–268 (2018). 29242887 10.1039/c7cs00607a

[12] Murray, C. I. & Van Eyk, J. E. Chasing cysteine oxidative modifications: proteomic tools for characterizing cysteine redox status. *Circ. Cardiovasc. Genet.* **5**, 591 (2012). 23074338 10.1161/CIRCGENETICS.111.961425 PMC3500588

[13] van der Reest, J., Lilla, S., Zheng, L., Zanivan, S. & Gottlieb, E. Proteome-wide analysis of cysteine oxidation reveals metabolic sensitivity to redox stress. *Nat. Commun.* **9**, 1581 (2018). 29679077 10.1038/s41467-018-04003-3 PMC5910380

[14] Wani, R., Nagata, A. & Murray, B. W. Protein redox chemistry: post-translational cysteine modifications that regulate signal transduction and drug pharmacology. *Front. Pharm.* **5**, 224 (2014). 10.3389/fphar.2014.00224 PMC4186267 25339904

[15] Paulsen, C. E. & Carroll, K. S. Cysteine-mediated redox signaling: chemistry, biology, and tools for discovery. *Chem. Rev.* **113**, 4633–4679 (2013). 23514336 10.1021/cr300163e PMC4303468

[16] Chung, H. S., Wang, S. B., Venkatraman, V., Murray, C. I. & Van Eyk, J. E. Cysteine oxidative posttranslational modifications: emerging regulation in the cardiovascular system. *Circ. Res.* **112**, 382–392 (2013). 23329793 10.1161/CIRCRESAHA.112.268680 PMC4340704

[17] Jeong, J. et al. Novel oxidative modifications in redox-active cysteine residues. *Mol. Cell Proteom.* **10**, M110.000513 (2011). 10.1074/mcp.M110.000513 PMC3047142 21148632

[18] Zhong, Q. et al. Protein posttranslational modifications in health and diseases: functions, regulatory mechanisms, and therapeutic implications. *MedComm* **4**, e261 (2023). 37143582 10.1002/mco2.261 PMC10152985

[19] Zuo, J. et al. Redox signaling at the crossroads of human health and disease. *MedComm* **3**, e127 (2022). 35386842 10.1002/mco2.127 PMC8971743

[20] Day, N. J., Gaffrey, M. J. & Qian, W. J. Stoichiometric thiol redox proteomics for quantifying cellular responses to perturbations. *Antioxidants* **10**, 499 (2021). 33807006 10.3390/antiox10030499 PMC8004825

[21] Go, Y.-M. & Jones, D. P. The redox proteome. *J. Biol. Chem.* **288**, 26512–26520 (2013). 23861437 10.1074/jbc.R113.464131 PMC3772199

[22] Duan, J. et al. Stochiometric quantification of the thiol redox proteome of macrophages reveals subcellular compartmentalization and susceptibility to oxidative perturbations. *Redox Biol.* **36**, 101649 (2020). 32750668 10.1016/j.redox.2020.101649 PMC7397701

[23] Guo, J. et al. Resin-assisted enrichment of thiols as a general strategy for proteomic profiling of cysteine-based reversible modifications. *Nat. Protoc.* **9**, 64–75 (2014). 24336471 10.1038/nprot.2013.161 PMC4038159

[24] Xiao, H. et al. A quantitative tissue-specific landscape of protein redox regulation during aging. *Cell* **180**, 968–983.e924 (2020). 32109415 10.1016/j.cell.2020.02.012 PMC8164166

[25] Wojdyla, K., Williamson, J., Roepstorff, P. & Rogowska-Wrzesinska, A. The SNO/SOH TMT strategy for combinatorial analysis of reversible cysteine oxidations. *J. Proteom.* **113**, 415–434 (2015). 10.1016/j.jprot.2014.10.015 25449835

[26] Zhang, T. et al. Regulation of hyperoxia-induced neonatal lung injury via post-translational cysteine redox modifications. *Redox Biol.* **55**, 102405 (2022). 35872399 10.1016/j.redox.2022.102405 PMC9307955

[27] Stanhope, S. C. et al. Proteome-wide quantitative analysis of redox cysteine availability in the Drosophila melanogaster eye reveals oxidation of phototransduction machinery during blue light exposure and age. *Redox Biol.* **63**, 102723 (2023). 37146512 10.1016/j.redox.2023.102723 PMC10189289

[28] Meng, J. et al. Global profiling of distinct cysteine redox forms reveals wide-ranging redox regulation in C. elegans. *Nat. Commun.* **12**, 1415 (2021). 33658510 10.1038/s41467-021-21686-3 PMC7930113

[29] Yang, J., Gupta, V., Carroll, K. S. & Liebler, D. C. Site-specific mapping and quantification of protein S-sulphenylation in cells. *Nat. Commun.* **5**, 4776 (2014). 25175731 10.1038/ncomms5776 PMC4167403

[30] Lennicke, C. & Cochemé, H. M. Redox metabolism: ROS as specific molecular regulators of cell signaling and function. *Mol. Cell* **81**, 3691–3707 (2021). 34547234 10.1016/j.molcel.2021.08.018

[31] Menger, K. E. et al. Fasting, but not aging, dramatically alters the redox status of cysteine residues on proteins in Drosophila melanogaster. *Cell Rep.* **13**, 1285 (2015). 28873344 10.1016/j.celrep.2015.10.048 PMC5643521

[32] Wang, X. et al. Age-, sex- and proximal-distal-resolved multi-omics identifies regulators of intestinal aging in non-human primates. *Nat. Aging* **4**, 414–433 (2024). 38321225 10.1038/s43587-024-00572-9 PMC10950786

[33] Smith, A. & McCulloh, R. J. Hemopexin and haptoglobin: allies against heme toxicity from hemoglobin not contenders. *Front. Physiol.* **6**, 187 (2015). 26175690 10.3389/fphys.2015.00187 PMC4485156

[34] Satapati, S. et al. Mitochondrial metabolism mediates oxidative stress and inflammation in fatty liver. *J. Clin. Invest.* **125**, 4447–4462 (2015). 26571396 10.1172/JCI82204 PMC4665800

[35] Zhao, Y. et al. ROS signaling under metabolic stress: cross-talk between AMPK and AKT pathway. *Mol. Cancer* **16**, 79 (2017). 28407774 10.1186/s12943-017-0648-1 PMC5390360

[36] Cancemi, G., Cicero, N., Allegra, A. & Gangemi, S. Effect of diet and oxidative stress in the pathogenesis of lymphoproliferative disorders. *Antioxidants* **12**, 1674 (2023). 37759977 10.3390/antiox12091674 PMC10525385

[37] Ragusa, A. Secondary metabolites for the reduction of oxidative stress. *Molecules* **28**, 7555 (2023). 38005277 10.3390/molecules28227555 PMC10673449

[38] Hidalgo, F. J., Nogales, F. & Zamora, R. Changes produced in the antioxidative activity of phospholipids as a consequence of their oxidation. *J. Agric. Food Chem.* **53**, 659–662 (2005). 15686416 10.1021/jf0483220

[39] Petitprez, F. et al. The murine microenvironment cell population counter method to estimate abundance of tissue-infiltrating immune and stromal cell populations in murine samples using gene expression. *Genome Med.* **12**, 86 (2020). 33023656 10.1186/s13073-020-00783-w PMC7541325

[40] Asano, K. et al. Intestinal CD169(+) macrophages initiate mucosal inflammation by secreting CCL8 that recruits inflammatory monocytes. *Nat. Commun.* **6**, 7802 (2015). 26193821 10.1038/ncomms8802 PMC4518321

[41] Yan, Y. X. et al. Artemisinin analogue SM934 ameliorates DSS-induced mouse ulcerative colitis via suppressing neutrophils and macrophages. *Acta Pharm. Sin.* **39**, 1633–1644 (2018). 10.1038/aps.2017.185 PMC6289314 29849131

[42] Ignacio, A. et al. Small intestinal resident eosinophils maintain gut homeostasis following microbial colonization. *Immunity* **55**, 1250–1267.e1212 (2022). 35709757 10.1016/j.immuni.2022.05.014

[43] Noubade, R. et al. NRROS negatively regulates reactive oxygen species during host defence and autoimmunity. *Nature* **509**, 235–239 (2014). 24739962 10.1038/nature13152

[44] Sawayama, Y. et al. Expression of myeloperoxidase enhances the chemosensitivity of leukemia cells through the generation of reactive oxygen species and the nitration of protein. *Leukemia* **22**, 956–964 (2008). 18273043 10.1038/leu.2008.8

[45] Mizuki, K. et al. Functional modules and expression of mouse p40(phox) and p67(phox), SH3-domain-containing proteins involved in the phagocyte NADPH oxidase complex. *Eur. J. Biochem.* **251**, 573–582 (1998). 9490028 10.1046/j.1432-1327.1998.2510573.x

[46] Strohecker, A. M. et al. Identification of 6-phosphofructo-2-kinase/fructose-2,6-bisphosphatase as a novel autophagy regulator by high content shRNA screening. *Oncogene* **34**, 5662–5676 (2015). 25772235 10.1038/onc.2015.23 PMC4573377

[47] Ganner, A. et al. The acetyltransferase p300 regulates NRF2 stability and localization. *Biochem. Biophys. Res. Commun.* **524**, 895–902 (2020). 32057361 10.1016/j.bbrc.2020.02.006

[48] Yan, J. et al. GPSM1 impairs metabolic homeostasis by controlling a pro-inflammatory pathway in macrophages. *Nat. Commun.* **13**, 7260 (2022). 36434066 10.1038/s41467-022-34998-9 PMC9700814

[49] Colman, R. J. & Anderson, R. M. Nonhuman primate calorie restriction. *Antioxid. Redox Signal.* **14**, 229–239 (2011). 20698791 10.1089/ars.2010.3224 PMC3000242

[50] Hofer, S. J., Carmona-Gutierrez, D., Mueller, M. I. & Madeo, F. The ups and downs of caloric restriction and fasting: from molecular effects to clinical application. *EMBO Mol. Med.* **14**, e14418 (2022). 34779138 10.15252/emmm.202114418 PMC8749464

[51] Ducarmon, Q. R. et al. Remodelling of the intestinal ecosystem during caloric restriction and fasting. *Trends Microbiol.* **31**, 832–844 (2023). 37031065 10.1016/j.tim.2023.02.009

[52] Redman, L. M. et al. Metabolic slowing and reduced oxidative damage with sustained caloric restriction support the rate of living and oxidative damage theories of aging. *Cell Metab.* **27**, 805–815.e804 (2018). 29576535 10.1016/j.cmet.2018.02.019 PMC5886711

[53] Sohal, R. S. & Weindruch, R. Oxidative stress, caloric restriction, and aging. *Science* **273**, 59–63 (1996). 8658196 10.1126/science.273.5271.59 PMC2987625

[54] Kazemi, A., Speakman, J. R., Soltani, S. & Djafarian, K. Effect of calorie restriction or protein intake on circulating levels of insulin like growth factor I in humans: a systematic review and meta-analysis. *Clin. Nutr.* **39**, 1705–1716 (2020). 31431306 10.1016/j.clnu.2019.07.030

[55] Mattison, J. A. et al. Caloric restriction improves health and survival of rhesus monkeys. *Nat. Commun.* **8**, 14063 (2017). 28094793 10.1038/ncomms14063 PMC5247583

[56] Sohal, R. S. & Forster, M. J. Caloric restriction and the aging process: a critique. *Free Radic. Biol. Med.* **73**, 366–382 (2014). 24941891 10.1016/j.freeradbiomed.2014.05.015 PMC4111977

[57] Wang, Y. & Wang, C. Quantitative reactive cysteinome profiling reveals a functional link between ferroptosis and proteasome-mediated degradation. *Cell Death Differ.* **30**, 125–136 (2023). 35974250 10.1038/s41418-022-01050-8 PMC9883465

[58] Gaffrey, M. J., Day, N. J., Li, X. & Qian, W. J. Resin-assisted capture coupled with isobaric tandem mass tag labeling for multiplexed quantification of protein thiol oxidation. *J. Vis. Exp.*10.3791/62671 (2021). 10.3791/62671 PMC8828046 34223836

[59] Su, Z. et al. Global redox proteome and phosphoproteome analysis reveals redox switch in Akt. *Nat. Commun.* **10**, 5486 (2019). 31792197 10.1038/s41467-019-13114-4 PMC6889415

[60] Qu, Z. et al. Proteomic quantification and site-mapping of *S*-nitrosylated proteins using isobaric iodoTMT reagents. *J. Proteome Resm* **13**, 3200–3211 (2014). 10.1021/pr401179v PMC4084841 24926564

[61] Pimkova, K. et al. Quantitative analysis of redox proteome reveals oxidation-sensitive protein thiols acting in fundamental processes of developmental hematopoiesis. *Redox Biol.* **53**, 102343 (2022). 35640380 10.1016/j.redox.2022.102343 PMC9157258

[62] Fang, Z. et al. Mass spectrometry-cleavable protein N-terminal tagging strategy for system-level protease activity profiling. *J. Am. Soc. Mass Spectrom.* **33**, 189–197 (2022). 34928623 10.1021/jasms.1c00350

[63] Li, J. et al. TMTpro-18plex: the expanded and complete set of TMTpro reagents for sample multiplexing. *J. Proteome Resm.* **20**, 2964–2972 (2021). 10.1021/acs.jproteome.1c00168 PMC8210943 33900084

[64] Jomova, K. et al. Reactive oxygen species, toxicity, oxidative stress, and antioxidants: chronic diseases and aging. *Arch. Toxicol.* **97**, 2499–2574 (2023). 37597078 10.1007/s00204-023-03562-9 PMC10475008

[65] Sharifi-Rad, M. et al. Lifestyle, oxidative stress, and antioxidants: back and forth in the pathophysiology of chronic diseases. *Front Physiol.* **11**, 694 (2020). 32714204 10.3389/fphys.2020.00694 PMC7347016

[66] Roh, K. B., Jung, E., Park, D. & Lee, J. Fumaric acid attenuates the eotaxin-1 expression in TNF-α-stimulated fibroblasts by suppressing p38 MAPK-dependent NF-κB signaling. *Food Chem. Toxicol.* **58**, 423–431 (2013). 23707484 10.1016/j.fct.2013.05.020

[67] Ashrafian, H. et al. Fumarate is cardioprotective via activation of the Nrf2 antioxidant pathway. *Cell Metab.* **15**, 361–371 (2012). 22405071 10.1016/j.cmet.2012.01.017 PMC3314920

[68] Sullivan, L. B. et al. The proto-oncometabolite fumarate binds glutathione to amplify ROS-dependent signaling. *Mol. Cell* **51**, 236–248 (2013). 23747014 10.1016/j.molcel.2013.05.003 PMC3775267

[69] Zheng, L. et al. Fumarate induces redox-dependent senescence by modifying glutathione metabolism. *Nat. Commun.* **6**, 6001 (2015). 25613188 10.1038/ncomms7001 PMC4340546

[70] Xu, Q. et al. Metagenomic and metabolomic remodeling in nonagenarians and centenarians and its association with genetic and socioeconomic factors. *Nat. Aging* **2**, 438–452 (2022). 37118062 10.1038/s43587-022-00193-0

[71] Tian, H. et al. Precise metabolomics reveals a diversity of aging-associated metabolic features. *Small Methods* **6**, e2200130 (2022). 35527334 10.1002/smtd.202200130

[72] Ding, J. et al. A metabolome atlas of the aging mouse brain. *Nat. Commun.* **12**, 6021 (2021). 34654818 10.1038/s41467-021-26310-y PMC8519999

[73] Ulrich, K. & Jakob, U. The role of thiols in antioxidant systems. *Free Radic. Biol. Med.* **140**, 14–27 (2019). 31201851 10.1016/j.freeradbiomed.2019.05.035 PMC7041647

[74] Klomsiri, C., Karplus, P. A. & Poole, L. B. Cysteine-based redox switches in enzymes. *Antioxid. Redox Signal.* **14**, 1065–1077 (2011). 20799881 10.1089/ars.2010.3376 PMC3064533

[75] Xiao, W. & Loscalzo, J. Metabolic responses to reductive stress. *Antioxid. Redox Signal.* **32**, 1330–1347 (2020). 31218894 10.1089/ars.2019.7803 PMC7247050

[76] Shinmura, K. Effects of caloric restriction on cardiac oxidative stress and mitochondrial bioenergetics: potential role of cardiac sirtuins. *Oxid. Med Cell Longev.* **2013**, 528935 (2013). 23577224 10.1155/2013/528935 PMC3614061

[77] Sharma, A. & Singh, A. K. Molecular mechanism of caloric restriction mimetics-mediated neuroprotection of age-related neurodegenerative diseases: an emerging therapeutic approach. *Biogerontology* **24**, 679–708 (2023). 37428308 10.1007/s10522-023-10045-y

[78] Kern, L., Kviatcovsky, D., He, Y. & Elinav, E. Impact of caloric restriction on the gut microbiota. *Curr. Opin. Microbiol.* **73**, 102287 (2023). 36868081 10.1016/j.mib.2023.102287

[79] Anderson, R. M. & Weindruch, R. Metabolic reprogramming, caloric restriction and aging. *Trends Endocrinol. Metab.* **21**, 134–141 (2010). 20004110 10.1016/j.tem.2009.11.005 PMC2831168

[80] Surugiu, R. et al. Molecular mechanisms of healthy aging: the role of caloric restriction, intermittent fasting, mediterranean diet, and ketogenic diet—a scoping review. *Nutrients* **16**, 2878 (2024). 39275194 10.3390/nu16172878 PMC11397047

[81] Ma, J. et al. iProX: an integrated proteome resource. *Nucleic Acids Res.* **47**, D1211–d1217 (2019). 30252093 10.1093/nar/gky869 PMC6323926

[82] Chen, T. et al. iProX in 2021: connecting proteomics data sharing with big data. *Nucleic Acids Res.* **50**, D1522–d1527 (2022). 34871441 10.1093/nar/gkab1081 PMC8728291

