# Supplementary material for: Low-input redoxomics facilitates global identification of metabolic regulators of oxidative stress in the gut
Source: Signal Transduct Target Ther. 2025 Jan 8;10:8. doi: 10.1038/s41392-024-02094-7 (PMC11707242; doi:10.1038/s41392-024-02094-7)

*Supplementary Material*

**Low-input redoxomics facilitates global identification of metabolic regulators of oxidative stress in the gut**

Xina Xiao^1,†^, Meng Hu^1,†^, Li Gao^1,†^, Huan Yuan^1,†^, Baochen Chong^1,†^, Yu Liu^1^, Rou Zhang^1^, Yanqiu Gong^1^, Dan Du^2^, Yong Zhang^2^, Hao Yang^2^, Xiaohui Liu^3^, Yan Zhang^1^, Huiyuan Zhang^1^, Heng Xu^1^, Yi Zhao^4^, Wenbo Meng^5^, Dan Xie^1^, Peng Lei^1^, Shiqian Qi^1^, Yong Peng^1^, Tao Tan^6^, Yang Yu^7^, Hongbo Hu^1^, Biao Dong^1^, Lunzhi Dai^1,8,^*

^1^National Clinical Research Center for Geriatrics, State Key Laboratory of Biotherapy, West China Hospital, Sichuan University, Chengdu, 610041, China.

^2^Advanced Mass Spectrometry Center, Research Core Facility, Frontiers Science Center for Disease-related Molecular Network, NHC Key Lab of Transplant Engineering and Immunology, West China Hospital, Sichuan University, Chengdu, 610041, China.

^3^School of Life Sciences, Tsinghua University, Beijing, 100084, China.

^4^Department of Rheumatology and Immunology, West China Hospital, Sichuan University, Chengdu, 610041, China.

^5^The First School of Clinical Medicine, Lanzhou University, Lanzhou, 730030, Gansu, China.

^6^State Key Laboratory of Primate Biomedical Research, Institute of Primate Translational Medicine, Kunming University of Science and Technology, Kunming, Yunnan 650500, China.

^7^Beijing Key Laboratory of Reproductive Endocrinology and Assisted Reproductive Technology and Key Laboratory of Assisted Reproduction, Ministry of Education, Center for Reproductive Medicine, Department of Obstetrics and Gynecology, Peking University Third Hospital, Beijing 100191, China.

^8^Frontiers Medical Center, Tianfu Jincheng Laboratory, Chengdu, 610212, China.

^†^These authors contributed equally to this work.

*Correspondence: lunzhi.dai@scu.edu.cn (Dr. Lunzhi Dai)

This file includes:

Supplementary Fig. 1 to Supplementary Fig. 4

Source data of Fig.5

Source data of Fig.6


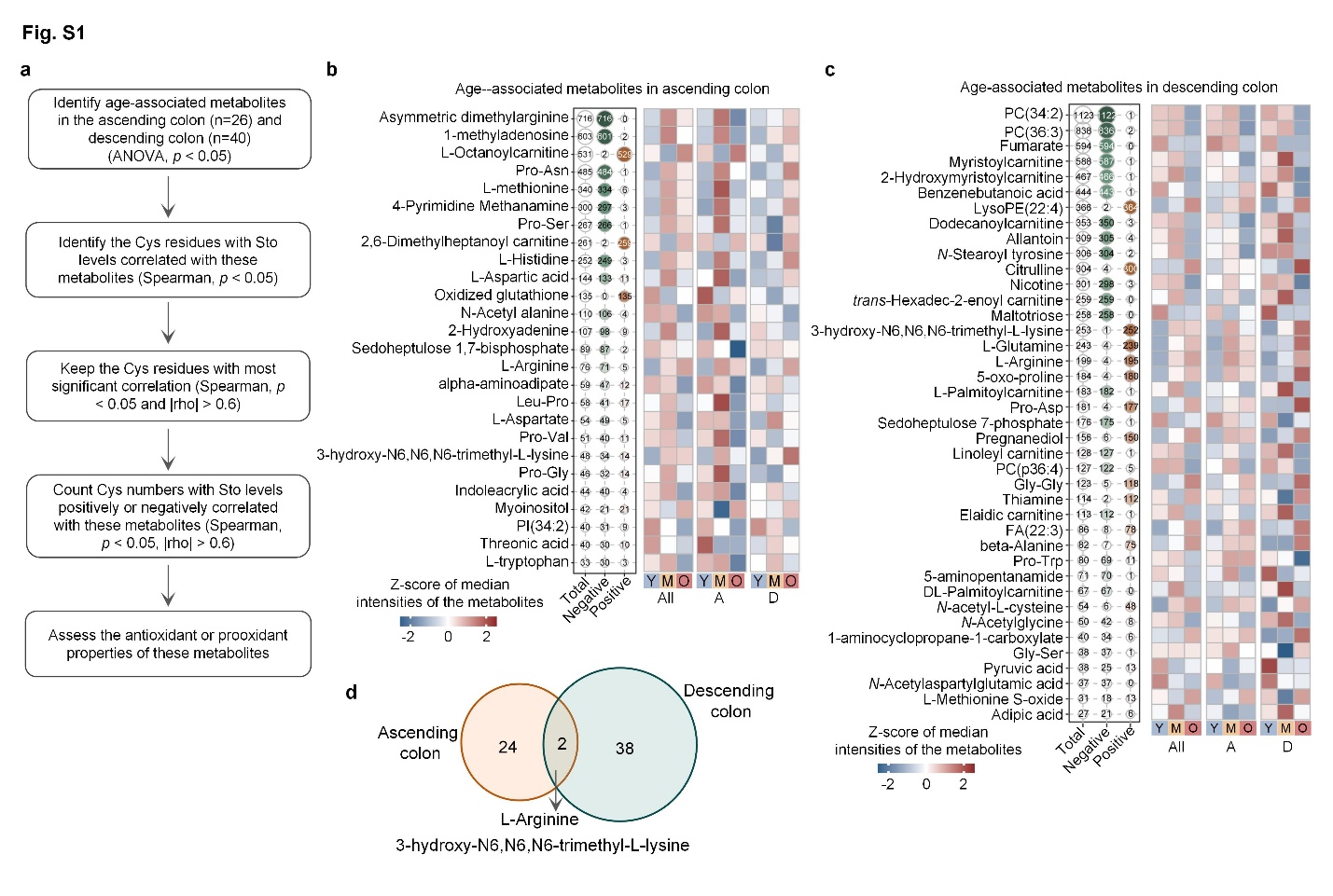


**Supplementary Fig. 1. Metabolic regulation of oxidative stress during aging in the ascending and descending colon.** **a,** Schematic illustrating the process of identifying metabolites influencing oxidative stress. **b,c,** List of metabolites significantly correlated with Sto levels in the ascending colon (**b**) and descending colon (**c**). Left: Bubble chart showing the number of Cys residues with Sto levels correlated with metabolites, both positively and negatively. Right: Heatmap displaying z-scored median metabolite abundances across groups. All, all samples irrespective of location effects; A, ascending colon; D, descending colon; Y, young; M, middle-aged; O, old. d, Venn diagram displaying the number of overlapping age-associated metabolites in the ascending colon and descending colon.


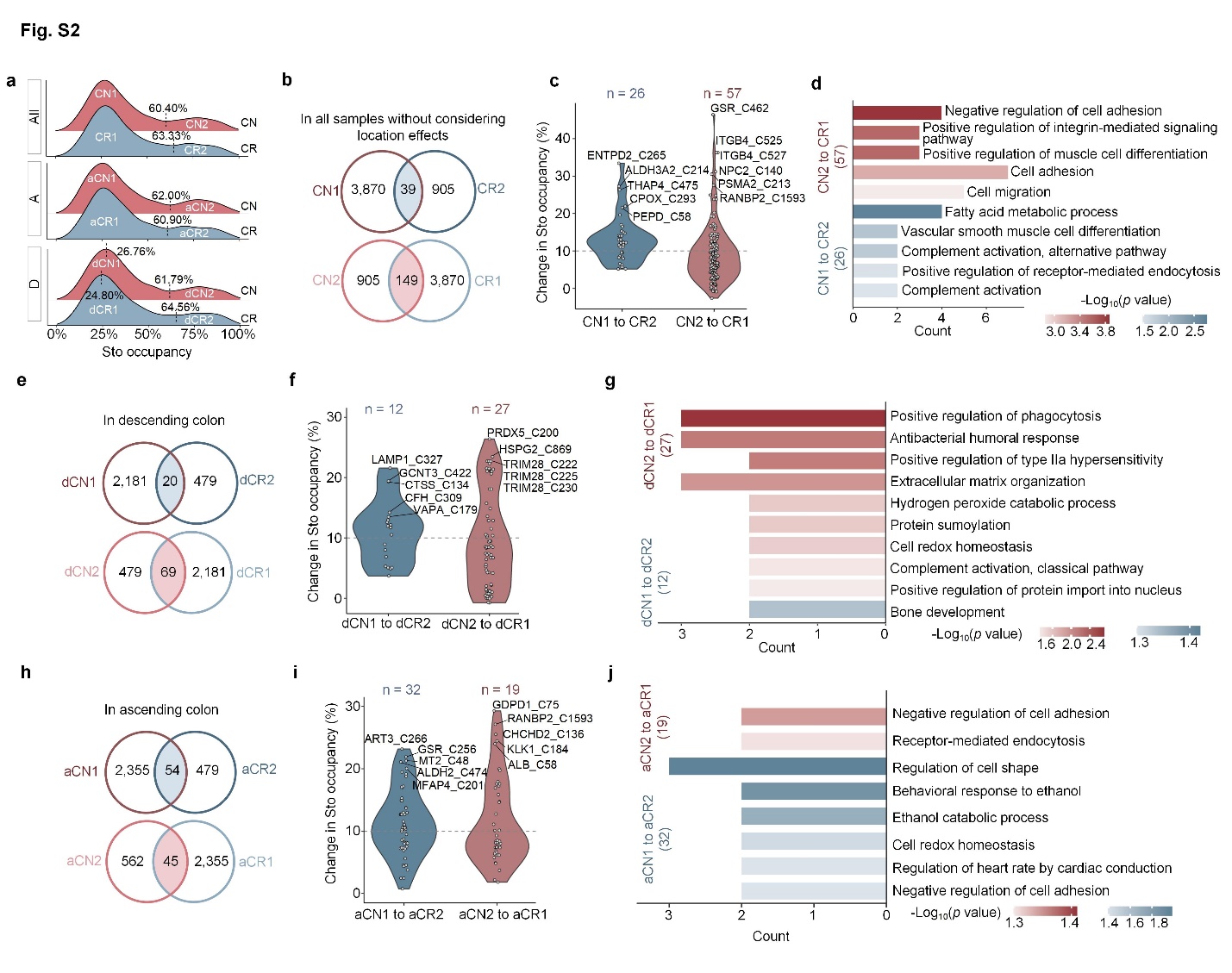


**Supplementary Fig. 2. Region-resolved bimodal characteristics of Cys oxidation occupancy in the colons of aged mice following CR intervention. a**, Density plots illustrating Sto occupancy distribution in all samples (All), ascending colon (A) and descending colon (D). The Sto occupancy displays a bimodal distribution, with peaks in the control (CN) group labeled as CN1 and CN2 for all samples, aCN1 and aCN2 for ascending colon, and dCN1 and dCN2 for descending colon, as well as peaks in the calorie restriction (CR) group marked as CR1 and CR2, aCR1 and aCR2 for ascending colon, and dCR1 and dCR2 for descending colon. The valley values between the two peaks are shown. **b-j**, Changes in Cys oxidation without considering location effects (**b-d**), or in the descending colon (**e-g**) or ascending colon (**h-j**) following CR intervention. The number of Cys residues with Sto occupancy transiting from CN1 to CR2 (blue shade) or from CN2 to CR1 (red shade) (**b**,**e**,**h**), as well as the number of Cys residues with Sto occupancy change greater than 10% was shown (**c,f,i**). Bar chart showing the enriched Gene Ontology Biological Process (GOBP) using proteins with Sto occupancy changes greater than 10% in all samples without considering location effects (**d**), in the descending colon (**g**) and in the ascending colon (**j**).


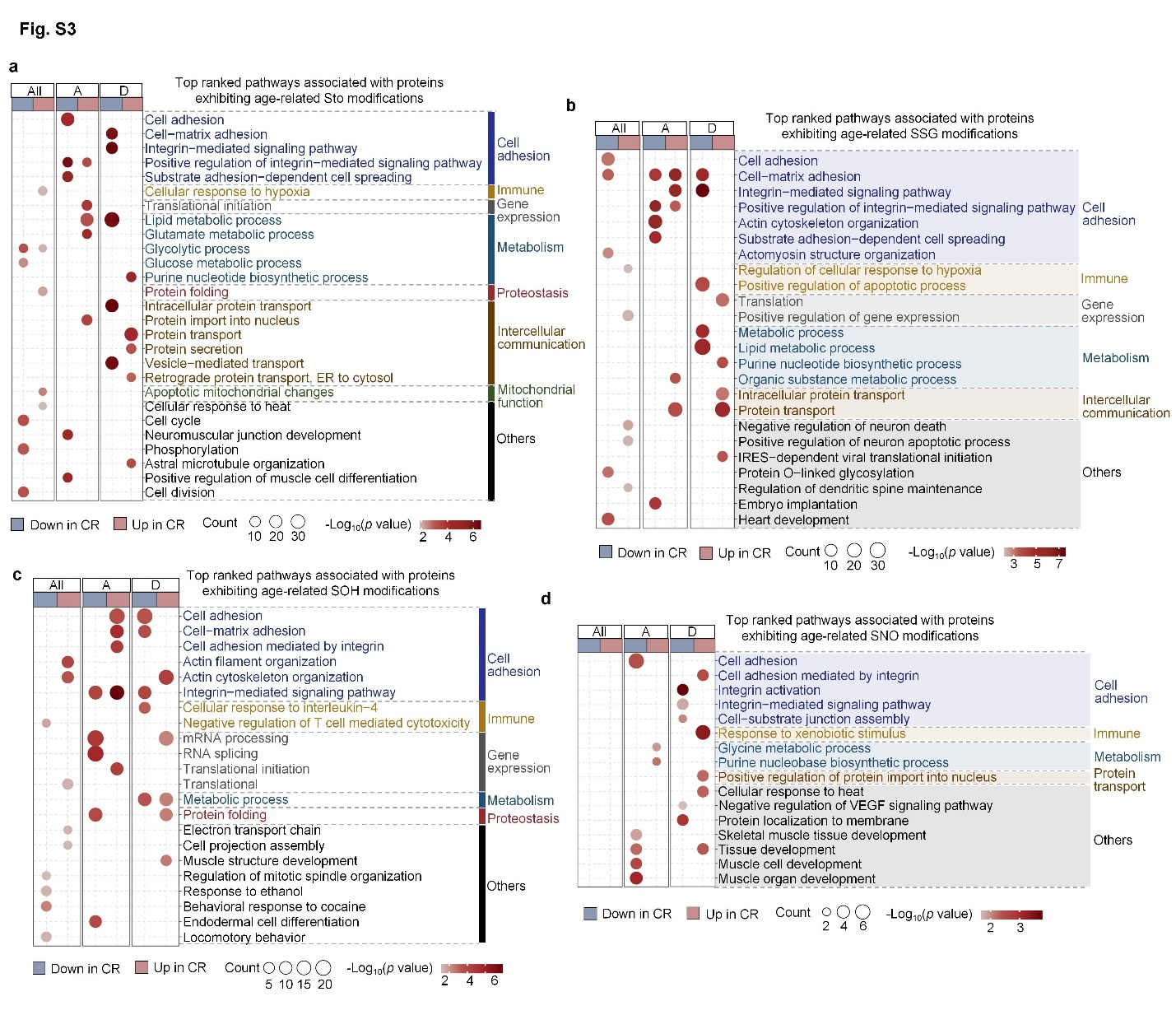


**Supplementary Fig. 3. Gene Ontology Biological Process (GOBP) analysis using proteins with significantly changed four modifications after CR interventions.** **a,** Sto modifications. **b,** SSG modifications. **c,** SOH modifications. **d,** SNO modifications. The top 5 enriched pathways in each group are shown. Circle size reflects the number of proteins involved in each pathway. Circle color represents -log_10_(*p* value). “All” denotes all samples without considering location effects, “A” means the ascending colon, and “D” represents the descending colon.


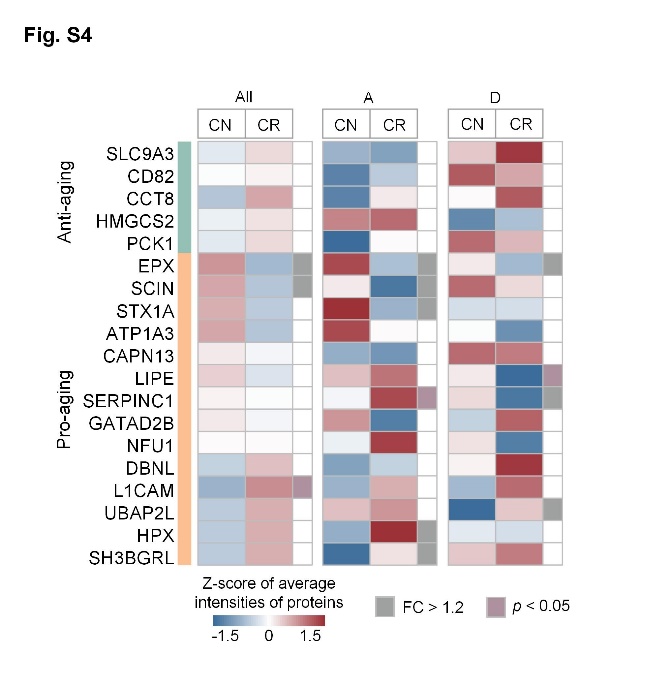


**Supplementary Fig. 4. Effects of CR on the expression of genes associated with intestinal aging in aged mice.** Annotation bars on the left denote anti-aging genes (green) and pro-aging genes (orange). The heatmap displays average protein expression levels of these genes in the control (CN) and calorie restriction (CR) groups. Deeper red indicates higher expression, while deeper blue indicates lower expression. Gray shading on the right side indicates protein expression fold changes between CN and CR groups greater than 1.2, and purple indicates significant protein expression changes after CR treatment (Student’s *t-*test, *p* < 0.05). "All" denotes all samples irrespective of location effects, "A" indicates samples from the ascending colon, and "D" represents samples from the descending colon.


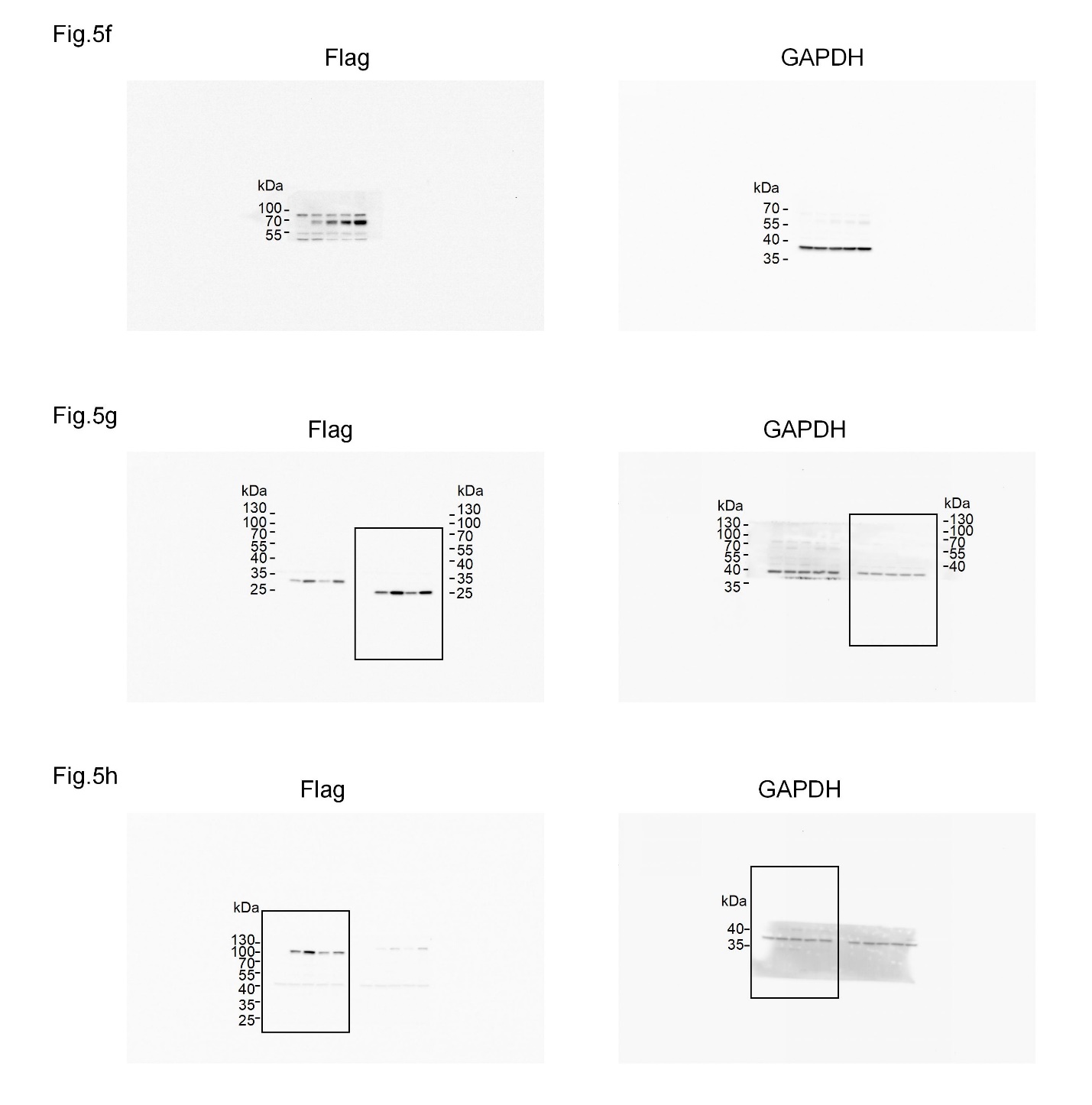


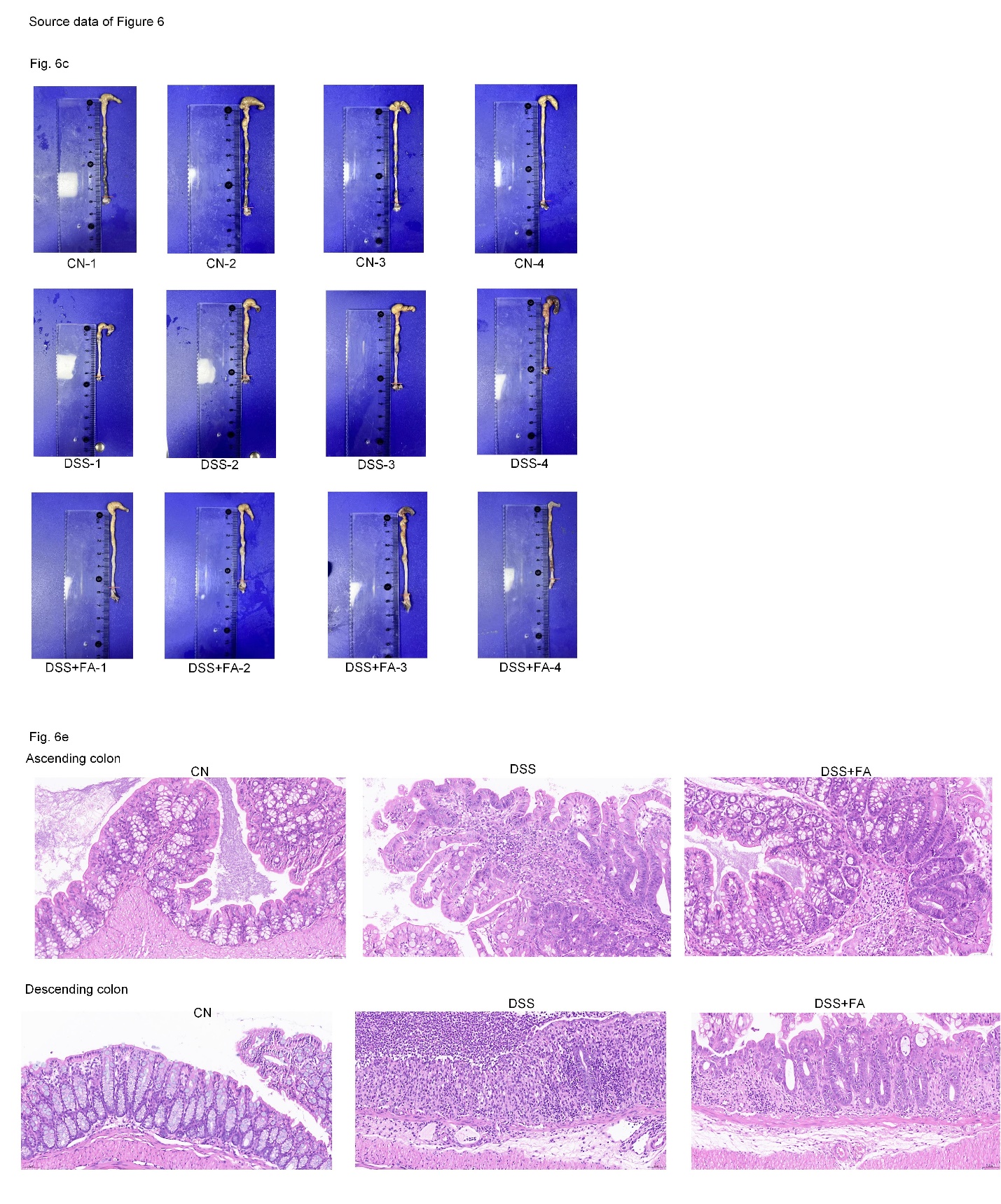

Supplement: Supplementary file 1 — SUPPLEMENTAL MATERIALS [file 41392_2024_2094_MOESM1_ESM.docx]
